# Effects of Th1/Th17 and Th2 cytokines on lipid metabolism in differentiated keratinocytes

**DOI:** 10.3389/fphys.2025.1387128

**Published:** 2025-02-19

**Authors:** Alessia Cavallo, Emanuela Camera, Miriam Maiellaro, Grazia Bottillo, Sarah Mosca, Daniela Kovacs, Enrica Flori, Giorgia Cardinali

**Affiliations:** Laboratory of Cutaneous Physiopathology, San Gallicano Dermatological Institute - IRCCS, Rome, Italy

**Keywords:** epidermal barrier, epidermal lipids, human keratinocytes cell line, 3D human epidermal equivalent, Th1/Th17 cytokines, Th2 cytokines

## Abstract

**Introduction:**

Abnormalities of keratinocyte differentiation and impairment of permeability barrier are features of inflammatory skin diseases driven by Th1/Th17 and Th2 immune response, such as psoriasis and atopic dermatitis. We aimed at identifying the signature of the Th1/Th17 and Th2 environments on keratinocytes, focusing on the expression of genes involved in the lipid metabolism and profiles of abundance of lipid metabolites.

**Methods:**

Human immortalized keratinocytes in prodifferentiative conditions induced by increasing calcium concentration, and 3D epidermal equivalents were treated with mixtures either of TNF-α and IL-17A plus Th1-related cytokines (IL-1α, IL-6) or of Th2 cytokines (IL-4, IL-13). The expression of genes involved in epidermal differentiation and lipid metabolism was evaluated by RT-PCR at 2, 4 and 7 days of treatment. The protein levels of early and late keratinocyte differentiation markers were assessed. The lipid composition was investigated by GCMS and LCMS.

**Results:**

Both Th1/Th17 and Th2 cytokine mixtures changed the expression of genes involved in the metabolism of fatty acids (FAs), i.e., FAS, FADS2, SCD1, and ALOX12B. Th1/ Th17 downregulated the ELOVL3 gene, which is implicated in the FAs elongation, while the mRNA levels of ABCA12 and HMGCR, genes involved in lipids transport and cholesterol synthesis, respectively, were decreased with both cytokine mixtures. DEGS1 and DEGS2, key enzymes in the ceramide synthesis, were downregulated and upregulated in the Th1/Th17 and Th2 environments, respectively. The mRNA expression of CERS3, which synthesizes ceramides containing long chain FAs, was increased by Th1/Th17 cytokines. Both Th1/ Th17 and Th2 cytokine mixtures lowered the CERS6 mRNA levels in differentiated keratinocytes. Effects specific to Th1/Th17 or Th2 cytokines were observed on freely extractable cell lipids. Th1/Th17 cytokines significantly inhibited the high calcium-induced synthesis of phospholipids (PCs, PEs, SMs), and short-chain ceramides, while the synthesis of ceramides with medium to long carbon chains was upregulated. Th2 cytokines caused a generalized decrement of free FAs, including long-chain ones. In contrast to 2D cultures, the 3D epidermal equivalents allowed the identification of altered profiles of acyland hexosyl-ceramides.

**Conclusion:**

The different effects exerted by Th1/Th17 and Th2 cytokines support, at least in part, the features of lipid barrier alterations specific to psoriasis or atopic dermatitis.

## Introduction

A complex cytokine environment underlies the onset and progression of skin inflammatory diseases, including psoriasis and atopic dermatitis (AD). Although these diseases manifest with specific clinical features, they present common aspects, like disruption of the epidermal permeability barrier (EPB) and infiltration and activation of T helper (Th) cells (Chen et al., 2022). Th1 and Th17 cells play a key pathogenic role in psoriasis through the release of several cytokines including tumor necrosis factor alpha (TNF-α), and IL-17A, which, in turn, trigger the release of IL-6 and IL-1α by keratinocytes. The Th2-type cytokines IL-4 and IL-13 are primarily implicated in AD pathogenesis (Bieber, 2020). Keratinocytes are the major cell constituents of the epidermis serving both structural and immune functions (Jiang et al., 2020). Cytokine-induced abnormalities in the keratinocyte differentiation process are linked to the alteration of cutaneous homeostasis and to the impairment of the EPB function (Huang et al., 2022). In the pathogenesis of AD it is unclear whether the abnormal immune response precedes or follows the perturbation of the EPB. Mutations in the filaggrin (FLG) gene, reduced expression of structural epidermal proteins, and lipid abnormalities in response to Th2 inflammatory cytokines are all linked to a dysfunctional EPB (Weidinger et al., 2018; Bieber, 2020). Psoriasis begins with the activation of the immune system in response to environmental triggers and/or dysregulated immune tolerance. In psoriatic skin, the epidermal granular layer is reduced or almost absent, while the stratum corneum (SC) is enriched in keratinocytes with incomplete cornification. The epidermis appears thickened due to hyperplasia (Kok et al., 2023). A fine-tuned lipid composition and distribution are determinant of the proper EPB functionality. Lipid manufacture in keratinocytes is a highly orchestrated process accomplished along an articulated differentiation program (Chieosilapatham et al., 2021). Epidermal lipids (Weidinger et al., 2018) are composed by ceramides, free fatty acids (FFAs) and cholesterol in equimolar concentration (Kendall and Nicolaou, 2022). The composition and the lamellar organization of lipids in the EPB are altered in both AD and psoriasis. Lipidomics studies have supported the discovery of abnormal lipid composition in psoriatic skin (Li et al., 2016). Psoriatic keratinocytes show increased levels of multiple ceramide classes (NS, NP, AS, EOS) and decreased amounts of the NDS ceramide class compared to healthy controls (Luczaj et al., 2020). In AD, the content of ceramide species with long chain (more than 50 carbon atoms) in the NS and NDS classes is severely reduced, while species with shorter chain (less than 40 carbon atoms) present higher concentration level in the SC (Bhattacharya et al., 2019; Ishikawa et al., 2010). An increase in the relative abundance of unsaturated FFAs and decrease in C22-28 FFAs, which introduces a conformational disorder in the lipid matrix, has been described in the SC of lesional AD skin (Danso et al., 2017). An impairment of HMGCoA reductase activity and cholesterol synthesis contributes to the deterioration of the EPB function and the epidermal hyperproliferation in psoriasis (Kuwatsuka et al., 2021).

Calcium is a key regulator of keratinocyte differentiation *in vivo* and *in vitro*. Increased intracellular calcium levels trigger the recruitment of kinases and phospholipases, and, in turn, activate second messengers crucial for the differentiation program (Bikle et al., 2012; Xie et al., 2005). High concentration of calcium occurs primarily in the stratum granulosum (SG) (Bikle et al., 2012). Due to the pivotal role of calcium in the epidermal homeostasis and in the formation of the EPB, elevation of calcium concentration is commonly adopted to induce keratinocyte differentiation *in vitro* (Borowiec et al., 2013; Lamb and Ambler, 2013). The use of primary keratinocytes is preferred. However, it has drawbacks such as limited life span and heterogeneity because of donor-to-donor variability. In this study, we used the commercially available human keratinocyte cell line, Ker-CT, as a suitable system to reproduce the differentiation programme that occurs in primary keratinocytes upon elevation of calcium concentration (Smits et al., 2017; Reijnders et al., 2015; Beckert et al., 2019; Kovacs et al., 2020; Teshima et al., 2023). To validate the use of Ker-CT cells as a model, we compared their response to Th1/Th17 and Th2 cytokine stimulation in high calcium conditions with that obtained in a pool of human primary keratinocytes. We then analysed key markers of early and late keratinocyte differentiation and regulators of lipid metabolism. Nevertheless, the two-dimensional (2D) models are limited in reflecting the intricate structure and functions of the epidermis, including the EPB (Piasek et al., 2023). Thus, we used three-dimensional (3D) human epidermal equivalents (HEEs) to favour the EPB formation, which enabled us to investigate the interplay between immune signals conveyed by Th1/Th17 and Th2 cytokines and lipogenic pathways in an epidermal-like model.

## Materials and methods

### Materials

The immortalized human keratinocyte cell line Ker-CT (ATCC^®^ CRL-4048TM) was purchased from ATCC (Manassas, VA, United States). M154, human keratinocyte growth supplements (HKGS), L-glutamine (2 mM), penicillin (100 µ/mL), and streptomycin (100 μg/mL), trypsin/EDTA and D-PBS were purchased from Invitrogen Technologies (Monza, Italy). TNF-α, IL17A, IL-1α, IL-6, IL-4 and IL-13 were from Peprotech (Cranbury, NJ, United States). AurumTM Total RNA Mini kit, SYBR Green PCR Master Mix, Bradford reagent were from Bio-Rad (Milan, Italy). RevertAidTM First Strand cDNA synthesis kit was from Thermo Fisher Scientific (Monza, Italy). GAPDH antibody (G9545) (1:5000) was from Sigma-Aldrich (Milan, Italy). HSP70 antibody (SC24) and anti-filaggrin (FLG) antibody (sc-66192) were from Santa Cruz Biotechnology (Santa Cruz, CA, United States). The anti-involucrin (IVL) antibody (ab53112), and anti-cytokeratin 10 (K10) antibody (ab76318), anti-loricrin (LOR) antibody (ab85679) were purchased from Abcam (Cambridge, UK). Anti-SLC27A4 MBS1757573) was form MyBioSource (San Diego, CA, United States), anti-ELOVL3 (NBP276673) was from Novus Biologicals (Cambridge, UK). Amersharm ECL Western blotting Detection Reagent was from GE Healthcare (Buckinghamshine, UK). RIPA lysis buffer, broad spectrum protease inhibitor cocktail, and broad-spectrum phosphatase inhibitor cocktail were from Boster Biological Technology Co. (Pleasanton, CA, United States).

### Chemicals

HPLC/MS-grade acetonitrile, methanol and 2-propanol, were purchased from Biosolve (Chimie SARL, Dieuze, France; BV, Valkenswaard, Netherlands), while HPLC/MS-grade ethyl acetate was from Carlo Erba (Milan, Italy). UHPLC/MS-grade water was purchased from LiChrosolv by Merck (Darmstadt, Germany). Dimethyl sulfoxide (DMSO), the antioxidant butylhydroxytoluene (BHT) and the mobile phase modifiers ammonium fluoride (NH_4_F) and ammonium formate (NH_4_COOH) were purchased from Sigma Aldrich (Milan, Italy). The Q-TOF calibration solution was prepared in acetonitrile from Agilent Technologies Tuning mix (HP0321 solution, Agilent Technologies, CA, United States). Deuterated Ceramide LIPIDOMIX^®^ (PN 330713) Mass Spec Standard Solution, EquiSPLASH™ LIPIDOMIX^®^ (PN 330731) Mass Spec Standard Solution, N-palmitoyl-d31-D-erythro-sphingosine (d31-Cer16:0, MW 569) and deuterated cholesterol sulfate sodium salt (d7-CHS, MW 495) were purchased from Avanti Polar Lipids (Alabaster, Alabama, US). Deuterated cholesterol-2,2,3,4,4,6-d6 (d6-CH, MW 392) was purchased from Toronto Research Chemicals (Toronto, Ontario, Canada). Hexadecanoic-9,9,10,10,11,11,12,12,13,13,14,14,15,15,16,16,16-d17 acid (d17-PA, MW 273), glyceryl trihexadecanoate-d98 (d98TG 48:0, MW 906) and n-hexadecyl-1,1,2,2-d4 hexadecanoate-16,16,16-d3 (d7WE, MW 488) were purchased from CDN Isotopes Inc. (Pointe-Claire, Quebec, Canada). Additional information on internal standards is reported in Supplementary Table S1.

### Culture of keratinocytes and 3D human epidermal equivalents

The immortalized human keratinocyte cell line Ker-CT (CRL-4048™) from American Type Culture Collection (ATCC^®^, Manassas, VA, United States) and a pool of neonatal primary keratinocytes from three different donors were cultured in defined Medium 154 with HKGS, L-glutamine (2 mM), penicillin (100 µ/mL), and streptomycin (100 µg/mlL, and Ca2+ (0.1 mM) at 37 °C under 5% CO_2_. For routine cell cultivation, cells were passaged when they reached 70%–90% of confluence. Cells were plated in fresh medium, in accordance with the experimental design (Supplementary Material S1). The cells maintained in Medium 154 in low calcium condition (0.1 mM) were used as control. Keratinocytes were induced to differentiate by switching the concentration of calcium in the culture medium from low (0.1 mM) to high (1.8 mM). Cells were stimulated with the pro-inflammatory cytokines, Th1/Th17 and Th2 type, in the pro-differentiative culture conditions. The Th1/Th17 cytokines, TNF-α (5 ng/mL), and IL-17A (10 ng/mL) with the addition of IL-6 (5 ng/mL) and IL-1α (10 ng/mL), from PeproTech (Rocky Hill, NJ, United States) were added to the fresh medium. IL-4 (10 ng/mL) and IL-13 (10 ng/mL) were used to investigate the Th2-type stimulus on keratinocytes. The medium was replaced every alternate day. Cells were harvested 2-, 4-, and 7-days post stimulation for gene and protein expression and lipid analysis. The Ker-CT cell line was used to generate 3D human epidermal equivalents (HEEs) as previously described (Flori et al., 2024). Briefly, Ker-CT were seeded on cell culture inserts (Thermo Scientific, Roskilde, Denmark; 0.4 µm pore size), maintained submerged for 3 days in CnT-Prime Epithelial Culture Medium (CnT-PR) (CellnTEC, Bern, Switzerland), and switched to CnT-Prime 3D Barrier Medium (CnT-PR-3D) in an air-liquid condition for 12 days. Fresh medium was replaced every alternate day. Mixtures of Th1/Th17 or Th2 cytokines, were added during the last 5 days of air-liquid culture. HEEs samples were processed for lipidomic profile. For histological analysis samples were formalin-fixed, paraffin-embedded and hematoxylin and eosin (H&E) stained.

### RNA extraction and quantitative real-time RT-PCR

Total RNA was isolated from keratinocytes using the Aurum™ Total RNA Mini kit, according to the manufacturer’s instructions. Total RNA samples were stored at −80°C until use. Following DNAse I treatment, cDNA was synthesized using a mix of oligo-dT and random primers and RevertAid™ First Strand cDNA synthesis kit according to the manufacturer’s instructions. Real-time RT-PCR was performed in a total volume of 10 μL with SYBR Green PCR Master Mix and 200 nM concentration of each primer. Sequences of all primers used are shown in Supplementary Table S2. Reactions were carried out in triplicate using a CFX96 Real Time System (Bio-Rad Laboratories S. r.l.). Melt curve analysis was performed to confirm the specificity of the amplified products. The mRNA expression was normalized to the mRNA expression of *GAPDH* by the change in the Δ cycle threshold (ΔCt) method and calculated based on 2^−ΔCt^. Results of the differentiated keratinocytes cultured in high calcium concentration were expressed as the fold change (FC) of control (taken as 1-fold). Data derived from differentiated keratinocytes treated with Th1/Th17 and Th2 cytokines were expressed as the FC relative to the high calcium condition. Results were represented as the mean ± SD of three independent experiments.

### Western blot analysis

Cells were lysed in RIPA buffer supplemented with a protease/phosphatase inhibitor cocktail and then sonicated. Total lysates were centrifuged at 12.000 rpm for 10 min at 4°C and then stored at −80°C until analysis. Following spectrophotometric protein measurement, equal amounts of protein were resolved on acrylamide SDS-PAGE and transferred onto a nitrocellulose membrane (Amersham Biosciences, Milan, Italy). Protein transfer efficiency was checked with Ponceau S staining (Sigma-Aldrich). Membranes were first washed with water, blocked with EveryBlot Blocking Buffer (Bio-Rad Laboratories Srl, Milan, Italy) for 10 min at room temperature and then treated overnight at 4°C with primary antibodies, according to instructions. A secondary anti-mouse or anti-rabbit IgG HRP-conjugated antibodies were used. Antibody complexes were visualized using chemiluminescence (ECL) substrate. A subsequent hybridization with anti-GAPDH or anti-HSP70 was used as a loading control. Protein levels were quantified by measuring the optical densities of specific bands using UVITEC Imaging System (Cambridge, UK). Results were expressed as the fold change relative to low calcium control (taken as 1-fold). Data represented the mean ± SD of three independent experiments.

### Immunofluorescence analysis

Ker-CT cells and human primary keratinocytes were fixed either with 4% paraformaldehyde followed by 0.1% Triton X-100 to allow permeabilization or with cold methanol at −20°C. Cells were then incubated with the following primary antibodies: anti-K10, anti-LOR, anti-FLG, anti-SLC27A4 and anti-ELOVL3. The primary antibodies were visualized using goat anti-rabbit Alexa Fluor 555 conjugate and goat anti-mouse Alexa Fluor 488 conjugate antibodies (Thermo Fisher Scientific). Coverslips were mounted using ProLong Gold antifade reagent with DAPI (Invitrogen). The fluorescence signal was evaluated by recording stained images, using a CCD camera (Zeiss, Oberkochen, Germany).

### Lipid extraction and sample preparation

Cells monolayers and HEEs collected at 7 and at 5 days of treatment, respectively, were suspended in distilled water and frozen and thawed 3 times to crack cell membranes. After centrifugation, the amount of protein present in the cell lysate was determined by Bradford’s assay. The extraction was performed with water/methanol/chloroform (1/3.32/1.66 v/v/v) in presence of a mixture of deuterated internal standards, which included deuterated cholesterol, FAs, ceramides and phospholipids. BHT was added to prevent autoxidation.

After a centrifugation step, the upper phase was transferred into a vial and the lipid extract was dried under nitrogen flow and suspended in 200 µL chloroform/methanol 2/1 v/v prior to analysis. To perform gas chromatography (GC) separation coupled to mass spectrometry (MS) analysis (GCMS), 20 µL of the lipid extract were dried under nitrogen and derivatized with 40 µL of N,O-bis(trimethyl-silyl)-trifluoroacetamide (BSTFA) spiked with 1% trimethylchlorosilane (TCMS) in pyridine at 60 °C for 60 min. For High Performance Liquid Chromatography (HPLC) coupled to MS (LCMS) analysis, 10 µL of dissolved extract were diluted with 40 µL of 2-propanol.

### GCMS

GCMS analysis of lipid extracts enabled the determination of FFAs and cholesterol; in epidermal equivalent models, sterol-like compounds were also identified and semi-quantified. The instrument used was an 8890 GC System combined with the 5977B Series MSD single quadrupole (Agilent Technologies, CA, United States). The analysis was performed on the HP-5MS UI fused silica column, chemically bonded with a 5%-phenyl-methylpolysiloxane phase (30 m × 0.250 mm internal diameter x 0.25 µm film thickness (Agilent Technologies CA, United States). Carrier gas (helium) flow rate was set at 1.2 mL/min. The GC oven program started at 80°C, reaching 280°C in 33 min and 310°C to final run time of 49 min. Samples were acquired in scan mode following EI (Ludovici et al., 2018) using MassHunter GC/MSD 5977B acquisition software (version 3.1.199). Data were processed by means of MassHunter Workstation Software Quantitative Analysis (version 10.1).

### HPLC

HPLC assisted the separation of a wide range of lipid species differing in molecular weight and polarity. Reversed Phase-HPLC (RP-HPLC) was applied to the separation of cholesterol sulfate (CHS), ceramides, glucosylceramides alias hexosylceramides (HexCers), and long chain FFAs (27–30 carbon atoms) in negative ESI (-ESI) ion mode, and of cholesterol esters (CEs), triglycerides (TGs) and diglycerides (DGs) in positive ion mode (+ESI). Hydrophilic Interaction Liquid Chromatography (HILIC) was used to separate and detect phospholipids, i.e., phosphatidylcholines (PCs), phosphatidylethanolamines (PEs) and sphingomyelins (SMs) in +ESI, and PEs, ether-linked phosphatidyl-ethanolamine (PE O-), phosphatidylinositols (PIs) and phosphatidylglycerols (PGs) in -ESI mode.

The chromatographic apparatus consisted of an Infinity II 1260 series HPLC equipped with a degasser, a quaternary pump, an autosampler and a column compartment (Agilent Technologies, CA, United States). RP-HPLC separation was conducted on a Zorbax Eclipse Plus C18 column (2.1 × 50 mm, 1.8 µm particle size, Agilent Technologies, CA, United States). The lipid extracts were eluted with a binary gradient of (A) 0.2 mM NH_4_F in water (18.2 Ω) and (B) 0.2 mM NH_4_F in methanol/2-propanol 80/20 (Maiellaro et al., 2024). The gradient elution was: 40% B, 0–2.0 min; 40%–99% B, 2.0–36.0 min, 99% B 36.0–46.0 min, 99%–40% B 46.0–48.0 min. A 10 min post-run time of 40% B was set for column re-equilibration. The column was thermostated at 60°C, the flow rate was 0.3 mL/min and the injection volume was 1 µL. HILIC separation was performed with a HALO HILIC column, 2.1 × 50 mm, 2.7 µm particle size (Advanced Materials Technology, AZ, United States), thermostated at 40°C. The mobile phase consisted of (A) 5 mM NH_4_COOH in water (18.2 Ω) and (B) acetonitrile. The flow rate was maintained at 0.4 mL/min during run time (22 min) and post-run time (10 min). The injection volume was 0.4 µL. Lipid extracts were eluted as follows: 98% B, 0–1.0 min; 98%–80% B, 1.0–18.0 min; 80% B, 18.0–20.0 min; 80%–98% B, 20.0–21.0 min, 98% B, 21.0–22.0 min.

### HRMS

Sample ionization was performed by the ESI Dual Agilent Jet Stream (AJS) interface connected to the 6,545 Quadrupole Time of Flight (Q-TOF) mass spectrometer (Agilent Technologies, CA, United States). Nitrogen was used for both nebulization and desolvation processes. The ion source gas temperature was set at 200°C and a flow rate of 12 L/min. Sheath gas temperature was set at 350°C; sheath gas flow rate was 12 L/min. The capillary voltage was 4000 V. The fragmentor and the skimmer voltage parameters were set at 120 V and 40 V, respectively. High Resolution MS (HRMS) acquisition was accomplished in full scan and auto MS/MS mode. The m/z range for MS and MS/MS was 59–1,700 at a mass resolving power of 40.000. For internal mass calibration and accurate mass measurement, a specific solution containing reference ions (m/z 121.0509 and m/z 922.0098 in +ESI; m/z 112.9856 and m/z 966.0007 in–ESI using NH_4_COOH; m/z 119.0363 and m/z 940.0015 in–ESI using NH_4_F) was vaporized by a second nebulizer in the spray chamber.

LCMS data were acquired using the MassHunter Data Acquisition Software (B.09.00, Agilent Technologies) and processed with Agilent MassHunter Workstation Profinder (version 10.0).

### Statistical analysis

Data derived from Western blot analysis and Real-Time RT-PCR were represented as mean ± SD of three independent experiments. The values were expressed as relative to the control (low calcium condition, set as 1). Statistical significance was assessed using paired Student’s t-test or ANOVA followed by Tukey’s multiple comparisons test using GraphPad Prism (GraphPad Software). The minimal level of significance was *p* < 0.05.

Mole amounts were derived by multiplying the area ratio of the analyte and the same class labelled ISTD by the pmol of the ISTD. Lipidomics data were calculated normalizing mole amounts by the protein content previously determined by Bradford’s assay.

Statistical analysis was performed by Agilent MassHunter Mass Profiler Professional (MPP) (version 15.1). A template (.csv) containing the final concentrations for the 107 annotated species in 2D keratinocyte lipid profiles expressed in pmol/mg protein was imported into the software. Samples were divided into groups according to the treatment, i. e., Ctr, ↑Ca^2+^, ↑Ca^2+^+Th1/Th17, ↑Ca^2+^+Th2 to determine significant differences of compounds across groups. The data in linear scale were log-transformed before performing statistical analysis. The log2 transformed concentration of lipid analytes is provided in Supplementary Table S3. An unpaired Student’s t-test was applied using Benjamini-Hochberg and false discovery rate (FDR) correction. The corrected p-value (p) and fold change (FC) cut-off were 0.05 and 1.5, respectively. Differences were considered statistically significant with *p* ≤ 0.05 and FC ≥ 1.5.

The values corresponding to the concentration expressed as pmol/mg protein of the 300 species data annotated in HEEs were log-transformed before performing statistical analysis. One-way analysis of variance (one-way ANOVA) was performed to determine relevant differences according to the treatment, i. e., Ctr, Th1/Th17, and Th2. Benjamini-Hochberg was used for multiple testing correction, and Tukey HSD as the *post hoc* test.

## Results

### Th1/Th17 and Th2 cytokines differently regulate keratinocyte differentiation process

To analyse the effects induced by Th1/Th17 and Th2 cytokines on the expression of genes related to the calcium-induced differentiation process, the immortalized human keratinocyte cell line Ker-CT was exposed to mixtures of Th1 (TNF-α)/Th17 (IL-17A) plus IL-6 and IL-1α, or Th2 (IL-4, IL-13) cytokines for 2, 4, and 7 days (Supporting information). A time-dependent increase in gene expression of *K10, FLG, LOR, CASP 14, IVL*, and *TGM1* was observed (Supplementary Figure S1). Th1/Th17 cytokines induced a significant decrease of the *K10* expression at 4 and 7 days. The Th2 cytokines increased *K10* expression only at 7 days. The induced expression of *FLG* remained unaffected following both Th1/Th17 and Th2 challenges. In contrast, both Th1/Th17 and Th2 cytokine mixtures decreased the *LOR* and *CASP14* mRNAs. The early effects on *LOR* mRNA expression perdured up to 7 days, while effects on *CASP14* mRNA were observed exclusively at the latest time point. The induction of *IVL* and *TGM1* mRNA expression during differentiation was further increased by Th1/Th17, while it was decreased by Th2 cytokines (Supplementary Figure S1).

**FIGURE 1:**
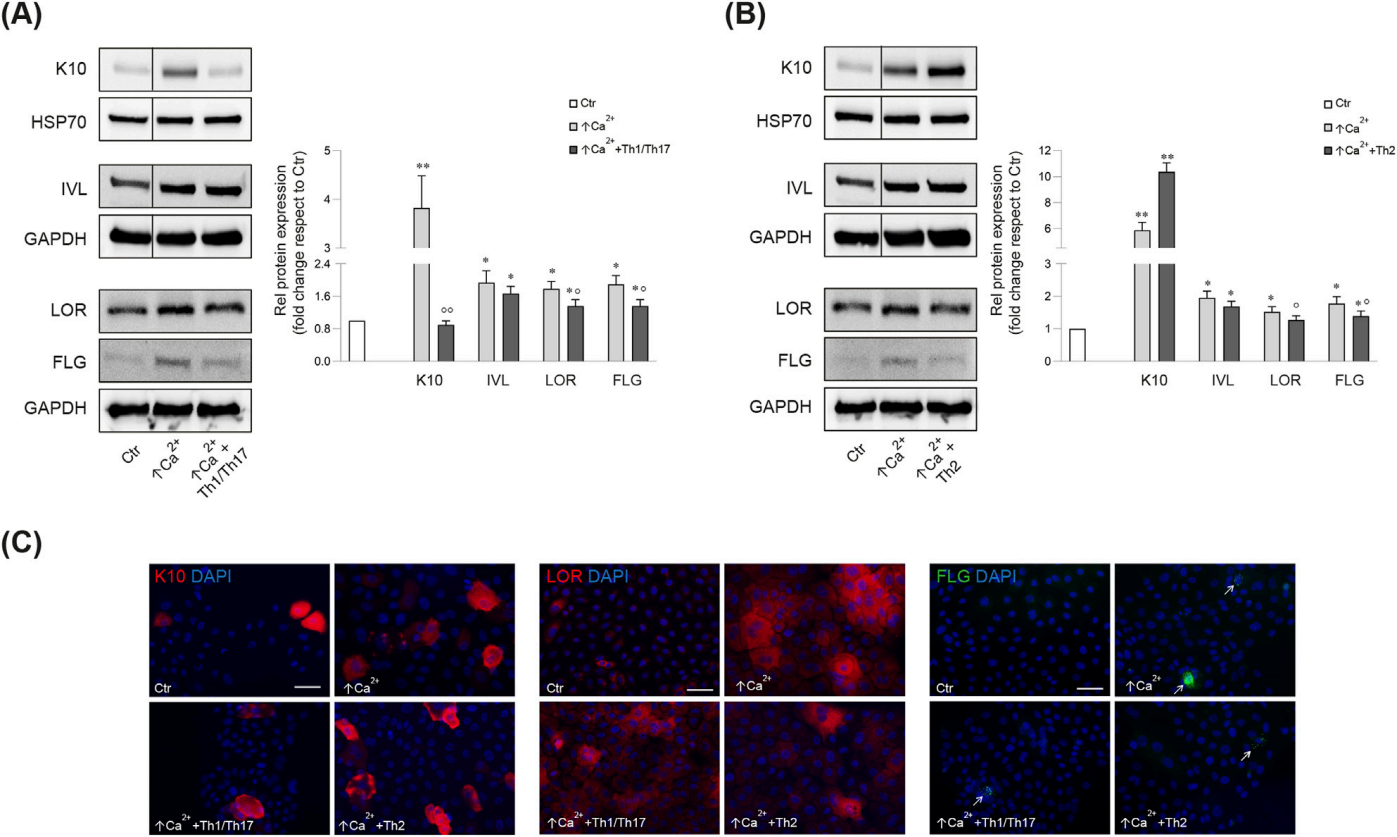
Western blot and corresponding densitometric analysis of K10, IVL, LOR, FLG (monomer, 37 kDa) expression in Ker-CT cells in differentiating conditions with ↑Ca^2+^ and after treatment with Th1/Th17 **(A)** and Th2 **(B)** cytokines at 7 days. GAPDH or HSP70 were used as the endogenous loading controls. Results are expressed as the fold change of the low calcium control cells (Ctr). Data represent the mean ± SD of three independent experiments (significance vs. low calcium control or vs. stimulated cells with ↑Ca^2+^ are marked with * and °, respectively; *p < 0.05, **p < 0.01 vs. low calcium cells; °p < 0.05, °p < 0.01 vs. differentiated cells). **(C)** Parallel immunofluorescence analysis of K10, LOR and FLG following stimulation with Th1/Th17 or Th2 cytokine types. Nuclei are counterstained in DAPI. Arrows point at FLG positive cells. Scale bar: 50 μm.

Consistent with the mRNA data, the Th1/Th17 cytokines reverted the calcium-induced K10 protein expression (Figures 1A, C), while, no significant change was observed after Th2 cytokines (Figures 1B, C). IVL protein expression was not affected by stimulation with both cytokine mixtures (Figures 1A, B). In contrast, both cytokine types counteracted the calcium-induced LOR and FLG protein expression (Figures 1A–C).

To validate the use of Ker-CT as a suitable model for studying the effects of Th1/Th17 and Th2 cytokines, we compared their response with that induced in human primary keratinocytes. Treatment of primary keratinocytes with the cytokine mixtures modulated the expression of the differentiation markers induced by high calcium condition in a fashion similar to that observed in Ker-CT cells (Supplementary Figure S2).

### Th1/Th17 and Th2 cytokines differentially regulate the expression of genes of lipid metabolism in differentiated keratinocytes

**FIGURE 2:**
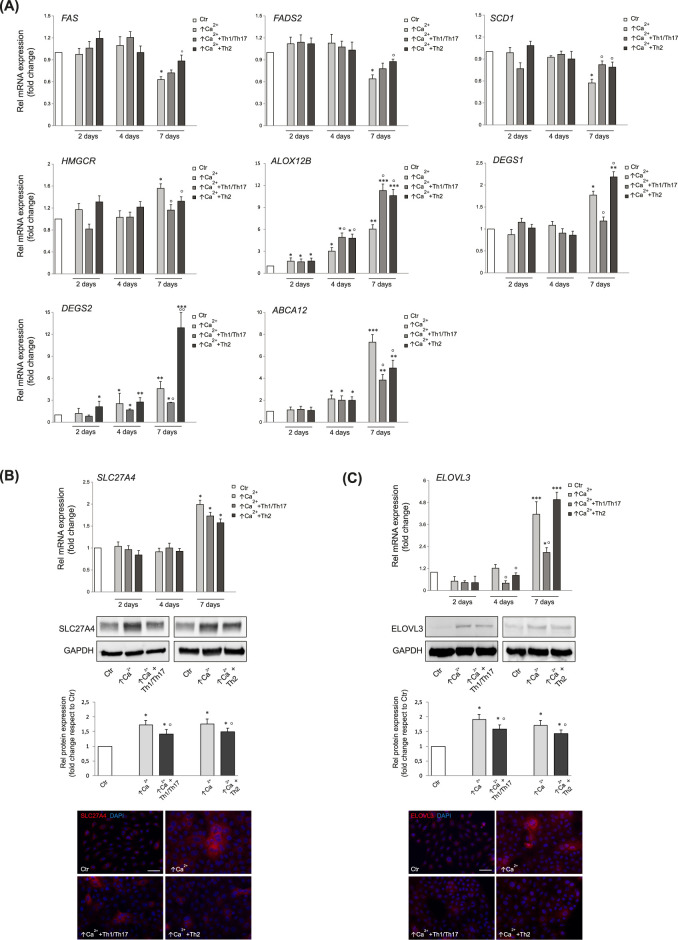
**(A)** Quantitative real time PCR analysis of genes involved in the synthesis of FFAs, ceramides and cholesterol in Ker-CT cells treated with ↑Ca^2+^, ↑Ca^2+^+Th1/Th17 and ↑Ca^2+^+Th2 for 2, 4 and 7 days. All mRNA values were normalized against the expression of *GAPDH* and were reported relative to low calcium control (Ctr). Data represent the mean ± SD of three independent experiments (significance vs. low calcium control or vs. stimulated cells with ↑Ca^2+^ are marked with * and °, respectively; *p < 0.05, **p < 0.01 and ***p < 0.001 vs. low calcium cells; °p < 0.05, °p < 0.01 vs. differentiated cells). Results are expressed as fold change relative to the value of cells grown in low calcium, which was set as 1 by definition. Quantitative real time PCR, Western blot with corresponding densitometry, and immunofluorescence analysis of **(B)** SLC27A4 and **(C)** ELOVL3 in Ker-CT cells in differentiating conditions with ↑Ca^2+^ and after treatment with Th1/Th17 and Th2 cytokines at 7 days. All mRNA values were normalized against the expression of *GAPDH* and were reported relative to low calcium control. Data represent the mean ± SD of three independent experiments (*p < 0.05, ***p < 0.001 vs. low calcium cells; °p < 0.05 vs. differentiated cells). Results are expressed as fold change relative to the value of cells grown in low calcium, which was set as 1 by definition. GAPDH was used as the endogenous loading controls for Western blot analysis. Results are expressed as the fold change respect to low calcium control cells. Data represent the mean ± SD of three independent experiments (*p < 0.05 vs. low calcium cells; °p < 0.05 vs. differentiated cells). Nuclei are counterstained with DAPI. Scale bar: 50 μm.

Genes of lipid metabolism were modulated in differentiated Ker-CT. *De novo* synthesis and desaturation of fatty acids (FAs) was repressed at 7 days in high calcium, as demonstrated by the lower mRNA levels of *FAS*, *FADS2*, and *SCD1* (Figure 2). Both Th1/Th17 and Th2 cytokine mixtures contrasted the decrease of *SCD1* mRNA levels, while only Th2 cytokines demonstrated to counteract the effects on *FAS* and *FADS2* mRNA expression induced in the differentiation process (Figure 2A).

The mRNA levels of *HMGCR*, gene of the mevalonate pathway in the cholesterol synthesis (Chen et al., 2023), significantly increased upon elevation of calcium concentration. Th1/Th17 and Th2 cytokines reverted the induction of HMGCR mRNA. ALOX12B plays a key role in the skin barrier function, serving oxidation of acyl-ceramides and protein crosslink (Meyer et al., 2023). Its mRNA level significantly increased in high calcium at all time points and was further augmented after treatment with both Th1/Th17 and Th2 cytokine mixtures at 4 and 7 days. *DEGS1*, responsible for the conversion of dihydroceramide into ceramide (Planas-Serra et al., 2023), significantly increased upon 7 days culture in differentiating conditions. Th1/Th17 cytokines decreased, whereas Th2 further increased *DEGS1* mRNA expression. Similar results were observed for the mRNA levels of *DEGS2*, a gene involved in the synthesis of sphingoid bases (Ota et al., 2023). In keratinocytes, ABCA12 serves the transmembrane lipid transport, particularly of ceramides, to form extracellular lipid layers (Proksch et al., 2008; Akiyama, 2014) and SLC27A4 (Schmuth et al., 2005) acts as a FFA trasporter. The mRNA levels of *ABCA12 and SLC27A4* significantly increased upon elevation of calcium concentration (Figure 2B). The rise of *ABCA12* levels started at 4 days and further increased at 7 days, while *SLC27A4* was increased at 7 days (Figure 2B). The treatment with both cytokine types significantly decreased the *ABCA12* mRNA expression at 7 days. The slight decrease of the *SLC27A4* mRNA concided with more pronounced effects on the protein expression (Figure 2B). Th1/Th17 stimulation reverted the prominent induction of both *ELOVL3* mRNA and protein levels at 7 days. Effects of Th2 cytokines were observed only on the reduction of ELOVL3 protein levels (Figure 2C). The Supplementary Table S4 reports the mean fold changes in the expression of the evaluated genes involved in the lipid metabolism. Analysis of key genes of the lipid metabolism in human primary keratinocytes after stimulation with Th1/Th17 and Th2 cytokines revealed a behaviour comparable to that of Ker-CT cells (Supplementary Figure S2), further supporting the suitability of the employed model.

### Different effects of Th1/Th17 and Th2 cytokines on the lipid profiles of differentiated keratinocytes

The effects of the Th1/Th17 and Th2 cytokines on the lipid profiles of differentiated keratinocytes at 7 days of treatment were studied by GCMS and LCMS. The two platforms together allowed for the determination of the abundance of 107 target species.

**FIGURE 3:**
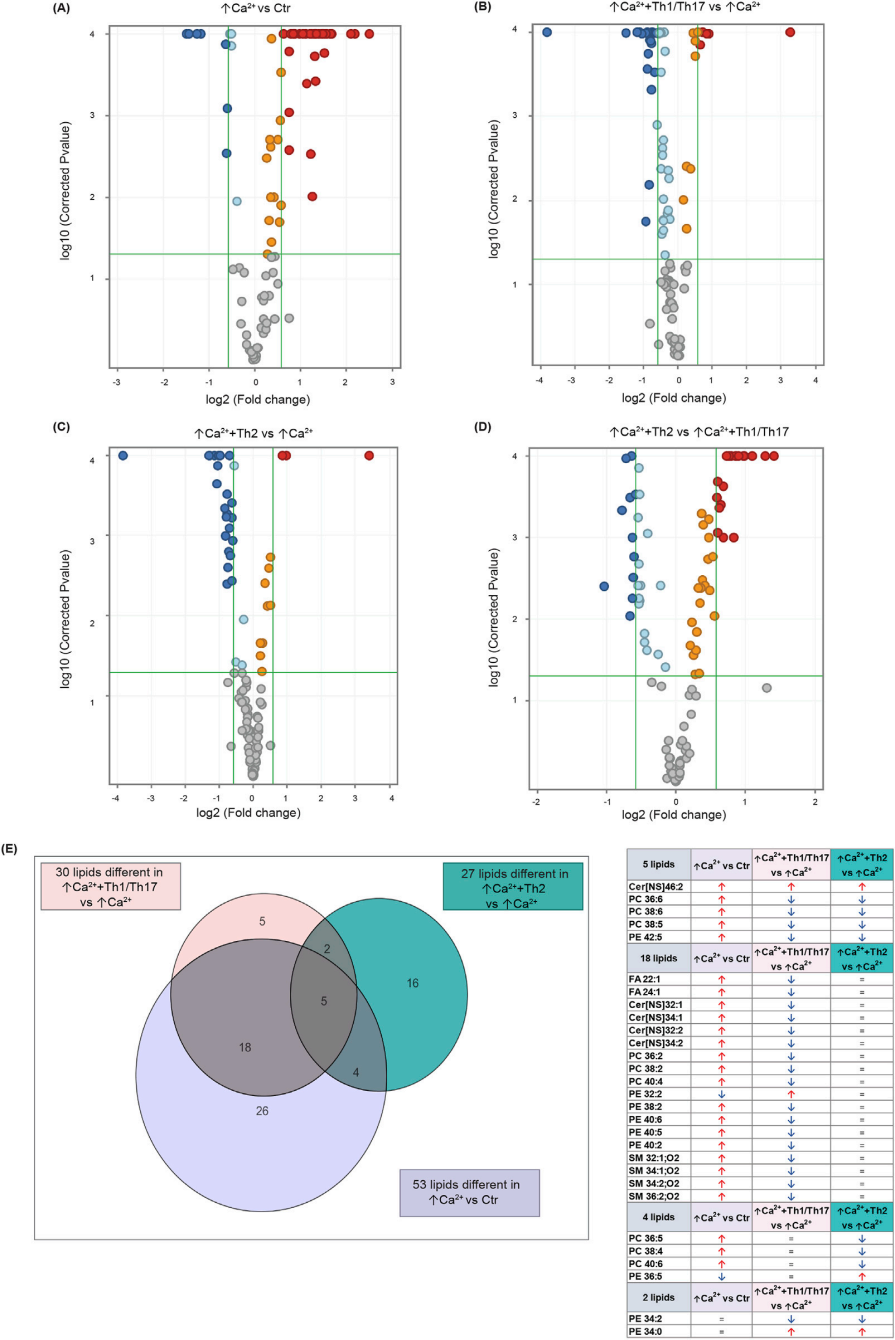
Results of the lipidomic analysis of Ker-CT cells under the different conditions at 7 days. Volcano plots depicting the following paiwise comparisons: **(A)** ↑Ca^2+^ vs. low calcium control (Ctr), **(B)** (↑Ca^2+^+Th1/Th17) vs. ↑Ca^2+^, **(C)** (↑Ca^2+^+Th2) vs. ↑Ca^2+^ **(D)** (↑Ca^2+^ +Th2) vs. ↑Ca^2+^ +Th1/Th17. Each dot corresponds to a lipid species: Blue and red dots indicate species that are statistically (p ≤ 0.05) decreased or increased (FC ≥ 1.5), respectively. Upregulated and downregulated species that did not pass the significance cut-off are in orange and light blue, respectively. Compounds that passed significance p ≤ 0.05 and FC ≥ 1.5 cut-offs are reported in Table 1. **(E)** Venn diagram and corresponding table showing the number of lipid species commonly and specifically modified by Th1/Th17 and Th2 cytokines.

Statistically significant differences were examined using pairwise comparisons. The volcano plot in Figure 3A shows the changes induced in the abundance of lipid species in high calcium differentiated keratinocytes. As reported in Table 1, 53 species were modulated at a statistically significant level (FC ≥ 1.5; p-value ≤0.05). The overall effect was an increase of lipid amounts in differentiated conditions. Specifically, 46 compounds were upregulated, i.e., cholesterol and CHS, most Cer [NS], including the short-chain Cer [NDS]34:0, some SMs, MUFAs, and PCs with 36–40 carbon atoms. Long-chain PEs (38–42 carbon atoms) were upregulated, while 7 short-chain PEs (31–36 carbon atoms) were downregulated. Overall, the presence of Th1/Th17 or Th2 cytokines modified the abundance of lipid species observed in high calcium concentration (Figures 3B, C). In differentiated keratinocytes exposed to Th1/Th17 cytokines, 25 and 5 species were significantly decreased and increased, respectively (Figure 3B). Th1/Th17 cytokines decreased the abundance of SMs, short-chain ceramides, FA 22:1, FA 24:1, PEs (except PE 32:2 and PE 34:0), and that of some PCs with different numbers of carbon atoms and double bonds (Table 2). The keratinocytes treated with Th2 cytokines showed a significant modulation of 27 lipid species (Figure 3C). These cytokines showed a suppressive effect, especially against FA synthesis. In fact, although minimally affected by differentiation, saturated FAs (SFAs) showed a strong decrease. Furthermore, Th2 cytokines caused the decrease of Cer [NS]46:1, highly unsaturated PCs, PE 34:2, and PE 42:5. The abundance of a few species, such as Cer [NS]46:2, PE 34:0 and PE 36:5, was upregulated (Table 3). Effects of Th1/Th17 and Th2 cytokines were compared in the volcano plot in Figure 3D, where 31 lipid species presented with significant different concentration (Table 4). The Th2 signal caused the upregulation of 20 compounds, i. e., CHS, FA 22:1, FA 24:1, short-chain ceramides, and some phospholipid. Eleven (11) compounds, i.e., 6 saturated FFAs, Cer [NS]42:1, Cer [NS]46:1 and 3 PCs, were downregulated. The Venn’s diagram in Figure 3E supports visualizing the effects consequent to Th1/Th17 and Th2 challenges compared to the high calcium condition. The table embedded in Figure 3 reports the individual lipid species at the intersection of the three subsets in the Venns’ diagram along the direction of their modification. The abundance of about half of the 53 species modulated by high calcium was modified by the cytokine environments. The majority of the 30 lipid species affected by the Th1/Th17 coincided with those characterizing the pro-differentiating conditions. In contrast only 9 out of the 27 species modified upon Th2 challenge, were common to the high calcium conditions. The Venn’s diagram shows that 5 species, i.e., Cer [NS]46:2, PC 36:6, PC 38:6, PC 38:5, and PE 42:5, were modulated in all three conditions. The number of compounds whose concentration was modified upon challenge of differentiated keratinocytes with Th1/Th17 or Th2 cytokines, was 18 and 4, respectively. Both Th1/Th17 and Th2 cytokine mixtures resulted in a decrease of PE 34:2 and an increase of PE 34:0 levels. The abundance of these compounds was not affected by differentiation alone. Further analyses were conducted to investigate the role of cholesterol in the *in vitro* system. CEs were detected in the keratinocytes lipid extracts in positive ion mode. MS data mining supported the presence of two CE species, i.e., CE (16:1) and CE (18:1). The abundance of both CE species was induced by high calcium at a significant extent. Th2 signals caused a significant increase in the concentration of CE (18:1) (Supplementary Figure S3).

**TABLE 1:** 53 Lipid species upregulated and downregulated in differentiated Ker-Ct compared to control (the cut-offs of fold change >1.5 and p ≤ 0.05).

Compound	p (Corr) ([↑Ca^2+^] Vs. [Ctr])	Regulation ([↑Ca^2+^] Vs. [Ctr])	Log FC ([↑Ca^2+^] Vs. [Ctr])
Cholesterol	1,643E-04	up	0.745
CHS	7,482E-08	up	2.504
FA 16:1	2,096E-05	up	0.856
FA 18:1	4,123E-04	up	1.130
FA 22:1	3,586E-07	up	1.581
FA 24:1	5,452E-07	up	1.459
FA 26:1	9,851E-06	up	0.849
Cer [NS]32:1	1,055E-07	up	1.526
Cer [NS]34:1	5,815E-07	up	1.318
Cer [NS]36:1	5,678E-06	up	0.973
Cer [NS]44:1	8,077E-05	up	0.622
Cer [NS]32:2	5,452E-07	up	1.300
Cer [NS]34:2	2,639E-08	up	2.180
Cer [NS]36:2	5,788E-07	up	1.328
Cer [NS]38:2	9,117E-04	up	0.753
Cer [NS]40:2	2,699E-07	up	1.309
Cer [NS]44:2	7,809E-05	up	0.809
Cer [NS]46:2	1,690E-05	up	0.850
Cer [NDS]34:0	5,452E-07	up	1.097
PC 36:6	2,988E-03	up	1.224
PC 36:5	2,675E-03	up	0.754
PC 36:3	6,628E-05	up	1.116
PC 36:2	4,229E-06	up	1.484
PC 36:1	4,255E-05	up	1.242
PC 38:6	5,839E-05	up	1.329
PC 38:5	1,726E-04	up	1.517
PC 38:4	1,905E-04	up	1.316
PC 38:3	3,586E-07	up	1.639
PC 38:2	1,260E-06	up	1.672
PC 38:1	4,346E-06	up	1.213
PC 40:6	3,791E-04	up	1.326
PC 40:5	7,523E-07	up	1.401
PC 40:4	4,346E-06	up	1.661
PE 38:3	2,685E-05	up	0.877
PE 38:2	7,482E-08	up	1.308
PE 40:6	8,580E-06	up	0.882
PE 40:5	3,984E-06	up	1.215
PE 40:2	2,699E-07	up	1.483
PE 42:5	2,039E-07	up	1.563
SM 32:1; O2	2,888E-06	up	1.044
SM 34:1; O2	3,384E-05	up	0.783
SM 44:1; O2	1,810E-06	up	1.485
SM 34:2; O2	1,042E-07	up	2.095
SM 36:2; O2	9,801E-03	up	1.266
SM 40:2; O2	8,362E-05	up	0.833
SM 44:2; O2	4,525E-06	up	1.350
PE 31:2	5,788E-07	down	−1.173
PE 32:2	2,699E-07	down	−1.495
PE 32:1	7,482E-08	down	−1.251
PE 34:4	8,518E-07	down	−1.431
PE 34:3	2,889E-03	down	−0.609
PE 36:5	1,366E-04	down	−0.636
PE 36:4	8,218E-04	down	−0.590

**TABLE 2:** 30 Lipid species upregulated and downregulated in [↑Ca^2+^ +Th1/Th17] Vs. [↑Ca^2+^] (the cut-offs of fold change >1.5 and p ≤ 0.05).

Compound	p (Corr) ([↑Ca^2+^+Th1/Th17] Vs. [↑Ca^2+^])	Regulation ([↑Ca^2+^+Th1/Th17] Vs. [↑Ca^2+^])	Log FC ([↑Ca^2+^+Th1/Th17] Vs. [↑Ca^2+^])
Cer [NS]42:1	5,247E-05	up	0.739
Cer [NS]46:2	3,667E-06	up	0.924
Cer [NS]48:2	1,422E-04	up	0.609
PE 32:2	5,012E-06	up	0.786
PE 34:0	3,892E-11	up	3.287
FA 22:1	8,403E-05	down	−0.743
FA 24:1	9,350E-05	down	−0.680
Cer [NS]32:1	2,818E-06	down	−0.924
Cer [NS]34:1	9,142E-06	down	−0.832
Cer [NS]32:2	5,026E-05	down	−0.794
Cer [NS]34:2	5,693E-09	down	−1.520
PC 30:0	4,932E-04	down	−0.771
PC 36:6	3,169E-04	down	−0.713
PC 36:2	1,327E-04	down	−0.746
PC 38:6	7,488E-05	down	−0.972
PC 38:5	2,896E-04	down	−0.876
PC 38:2	1,278E-04	down	−0.839
PC 40:4	1,278E-04	down	−0.779
PE 34:2	6,107E-13	down	−3.851
PE 38:5	2,824E-05	down	−0.652
PE 38:2	1,291E-05	down	−0.703
PE 40:6	1,775E-04	down	−0.873
PE 40:5	2,824E-05	down	−0.916
PE 40:2	8,830E-06	down	−0.932
PE 42:5	3,582E-06	down	−1.096
SM 32:1; O2	1,587E-06	down	−1.006
SM 34:1; O2	4,954E-05	down	−0.594
SM 38:1; O2	1,726E-02	down	−0.898
SM 34:2; O2	3,296E-06	down	−1.131
SM 36:2; O2	6,519E-03	down	−0.845

**TABLE 3:** 27 Lipid species upregulated and downregulated in [↑Ca^2+^ +Th2] Vs. [↑Ca^2+^]. (the cut-offs of fold change >1.5 and p ≤ 0.05).

Compound	p (Corr) ([↑Ca^2+^+Th2] Vs. [↑Ca^2+^])	Regulation ([↑Ca^2+^+Th2] Vs. [↑Ca^2+^])	Log FC ([↑Ca^2+^+Th2] Vs. [↑Ca^2+^])
Cer [NS]46:2	2,238E-05	up	0.977
PE 34:0	2,831E-10	up	3,407
PE 36:5	2,238E-05	up	0.861
FA 12:0	5,207E-05	down	−1.270
FA 14:0	2,238E-05	down	−0.702
FA 15:0	5,363E-04	down	−0.771
FA 16:0	8,122E-04	down	−0.700
FA 17:0	4,055E-03	down	−0.766
FA 18:0	1,009E-03	down	−0.811
FA 23:0	1,369E-04	down	−1.043
FA 24:0	2,282E-04	down	−1.068
FA 25:0	4,585E-04	down	−0.827
FA 26:0	2,238E-05	down	−1.144
FA 27:0	2,490E-03	down	−0.743
FA 28:0	1,576E-03	down	−0.713
FA 29:0	3,686E-03	down	−0.631
FA 30:0	1,796E-03	down	−0.672
Cer [NS]46:1	5,971E-04	down	−0.629
PC 36:6	8,037E-06	down	−1.298
PC 36:5	3,033E-04	down	−0.766
PC 36:4	5,946E-04	down	−0.785
PC 38:6	2,238E-05	down	−1.021
PC 38:5	1,153E-03	down	−0.596
PC 38:4	1,063E-05	down	−0.974
PC 40:6	2,254E-05	down	−0.978
PE 34:2	2,831E-10	down	−3.830
PE 42:5	3,958E-04	down	−0.625

**TABLE 4:** 31 Lipid species upregulated and downregulated in [↑Ca^2+^ +Th2] Vs. [↑Ca^2+^ +Th1/Th17] (the cut-offs of fold change >1.5 and *p* ≤ 0.05).

Compound	p (Corr) ([↑Ca^2+^+Th2] Vs. [↑Ca^2+^+Th1/Th17])	Regulation ([↑Ca^2+^+Th2] Vs. [↑Ca^2+^+Th1/Th17])	Log FC ([↑Ca^2+^+Th2] Vs. [↑Ca^2+^+Th1/Th17])
CHS	3,994E-04	up	0.653
FA 22:1	8,548E-05	up	0.868
FA 24:1	7,326E-06	up	0.798
Cer [NS]32:1	6,104E-09	up	1,283
Cer [NS]34:1	6,070E-09	up	1,101
Cer [NS]32:2	8,513E-05	up	0.769
Cer [NS]34:2	6,070E-09	up	1.419
Cer [NS]38:2	2,406E-04	up	0.684
PC 30:0	1,005E-03	up	0.687
PC 36:2	4,373E-04	up	0.624
PC 38:2	3,271E-05	up	0.986
PC 38:1	2,057E-04	up	0.598
PC 40:4	2,217E-05	up	0.854
PE 36:2	3,292E-04	up	0.596
PE 38:2	8,663E-06	up	0.769
PE 40:5	8,890E-04	up	0.599
PE 40:2	8,663E-06	up	0.727
SM 32:1; O2	8,513E-05	up	0.977
SM 34:2; O2	2,528E-05	up	0.909
SM 36:2; O2	1,005E-03	up	0.840
FA 12:0	3,952E-03	down	−1.030
FA 15:0	3,292E-04	down	−0.656
FA 17:0	9,208E-03	down	−0.655
FA 23:0	4,673E-04	down	−0.777
FA 24:0	3,103E-03	down	−0.613
FA 26:0	1,005E-03	down	−0.627
Cer [NS]42:1	1,970E-05	down	−0.637
Cer [NS]46:1	1,091E-04	down	−0.721
PC 36:6	2,996E-04	down	−0.585
PC 38:4	1,710E-03	down	−0.602
PC 40:6	5,605E-03	down	−0.620

### The gene expression of ceramide synthases correlates with the abundance of the corresponding lipid products

**FIGURE 4:**
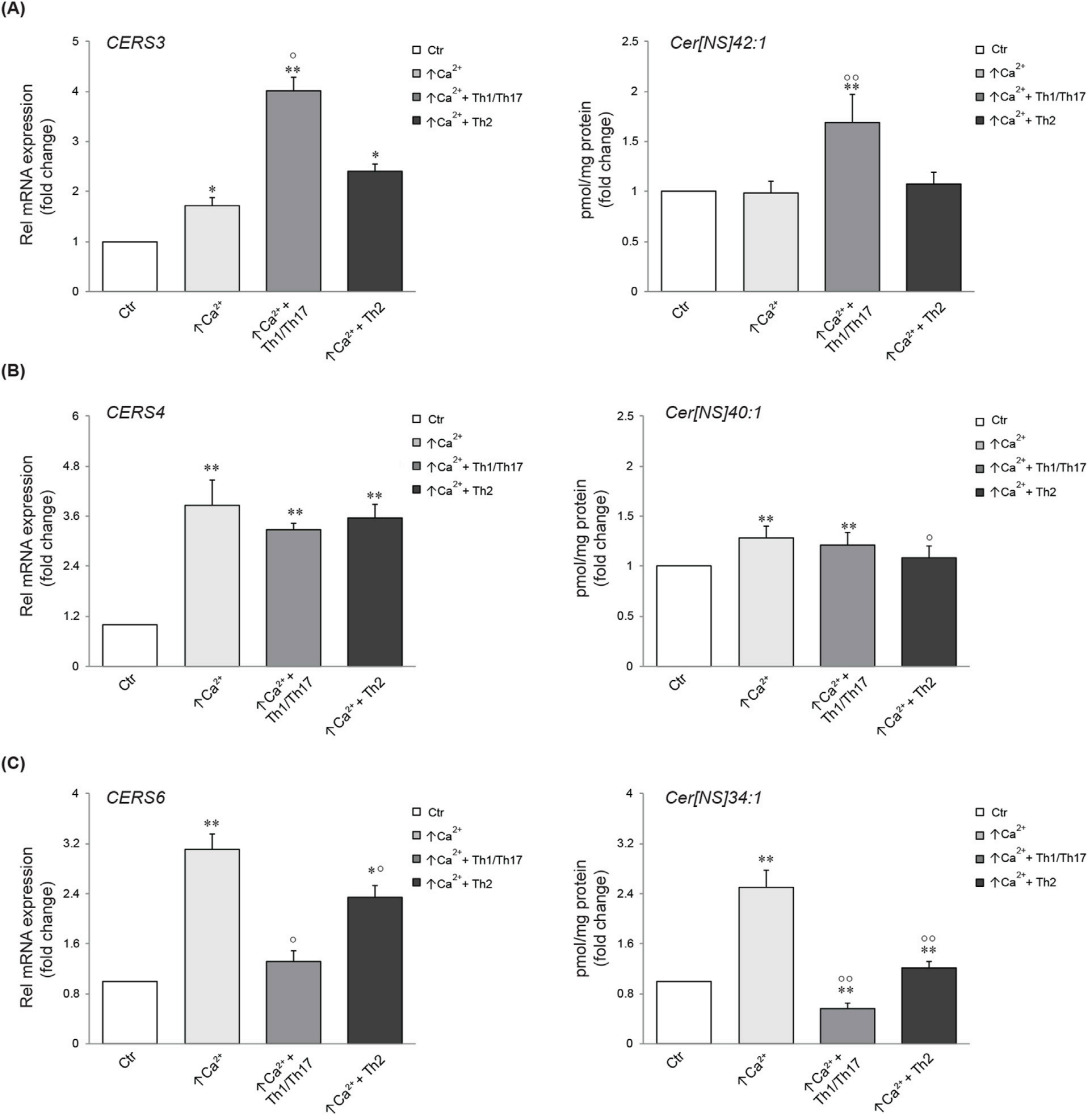
Comparison between gene expression and corresponding synthesized lipid species. Quantitative real time PCR analysis of *CERS3* **(A)**, *CERS4*, **(B)** *CERS6* **(C)** and respective products in differentiated Ker-CT cells, co-treated with Th1/Th17 or Th2, for 7 days. All mRNA values were normalized against the expression of *GAPDH* and were expressed relative to low calcium control cells (Ctr). The data are expressed as mean ± SD of three independent experiments (significance vs. low calcium control or vs. stimulated cells are marked with * and °, respectively; *p < 0.05 and **p < 0.01 vs. low calcium control; °p < 0.05 and °p < 0.01 vs. stimulated cells with high calcium).

We next investigated the association between the mRNA expression of specific ceramide synthesis-related enzymes and the levels of the corresponding lipids. Five types of ceramide synthase 2–6 (*CERS 2–6*) have been identified in human keratinocytes and are known to be involved in the skin barrier alteration associated with AD (Ito et al., 2017). Figure 4 displays the gene expression of *CERS3*, *4*, and *6*, which are involved in the synthesis of the Cer [NS] class, and the abundance profile of their corresponding member with specific FA chain lengths (Park et al., 2010; Cingolani et al., 2016). *CERS3* mRNA expression was promoted in differentiated keratinocytes. A pronounced increase was observed upon treatment with Th1/Th17 cytokines. The elevation of *CERS3* due to differentiation remained unchanged following Th2 challenge. The amount of the Cer [NS]42:1, which is the prominent product of *CERS3*, resulted unmodified by increasing calcium concentration, whereas it was significantly induced by Th1/Th17 stimulation (Figure 4A). The expression of *CERS4* augmented in response to high calcium and this effect was not modified by the co-treatment with both cytokine types. In contrast, Th2 stimulation decreased the concentration of the corresponding metabolite Cer [NS]40:1 compared to high calcium (Figure 4B). The comparison between *CERS6* and its product is depicted in Figure 4C. A significant elevation of *CERS6* mRNA and Cer [NS]34:1 concentration was observed in differentiated conditions compared to the control. This effect was counteracted by both Th1/Th17 and Th2 cytokine mixtures.

### Modulation of lipid profiles induced in 3D human epidermal equivalents by Th1/Th17 and Th2 cytokines

**FIGURE 5:**
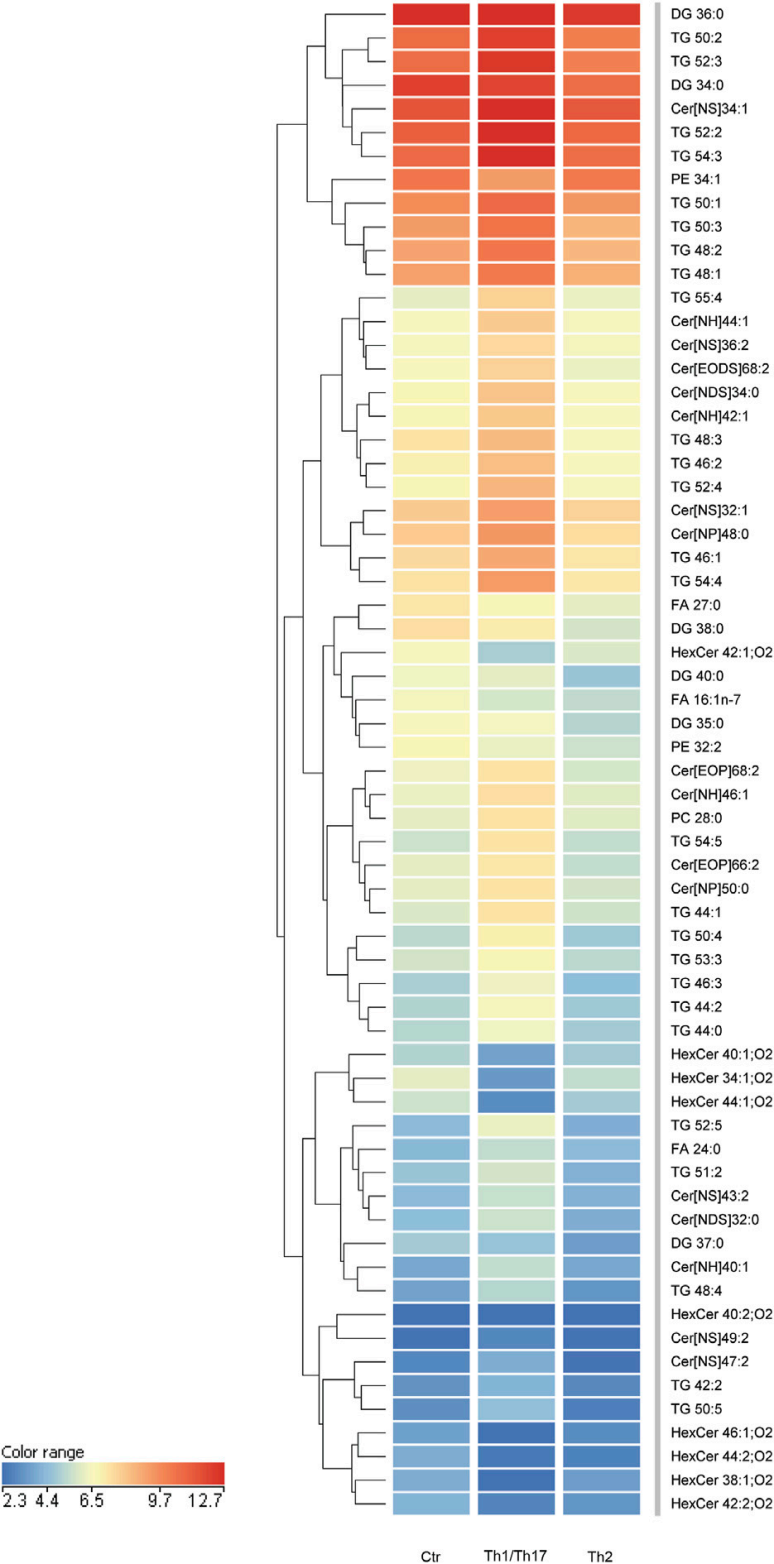
Hierarchical clustering of 64 lipid species, whose abundance was modulated upon treatment with Th1/Th17 or Th2 cytokines of 3D epidermal equivalents. The three columns in the heatmap represent the averaged data of six samples per experimental condition. The color intensity indicates the logarithmically transformed abundances of each lipid species, ranging from dark blue (lowest abundance) to red (highest abundance). The dendrogram supports the identification of shared behaviours and interrelationships among the lipids in the three conditions. The 64 lipid species are listed in cluster order in the Supplementary Table S6.

**FIGURE 6:**
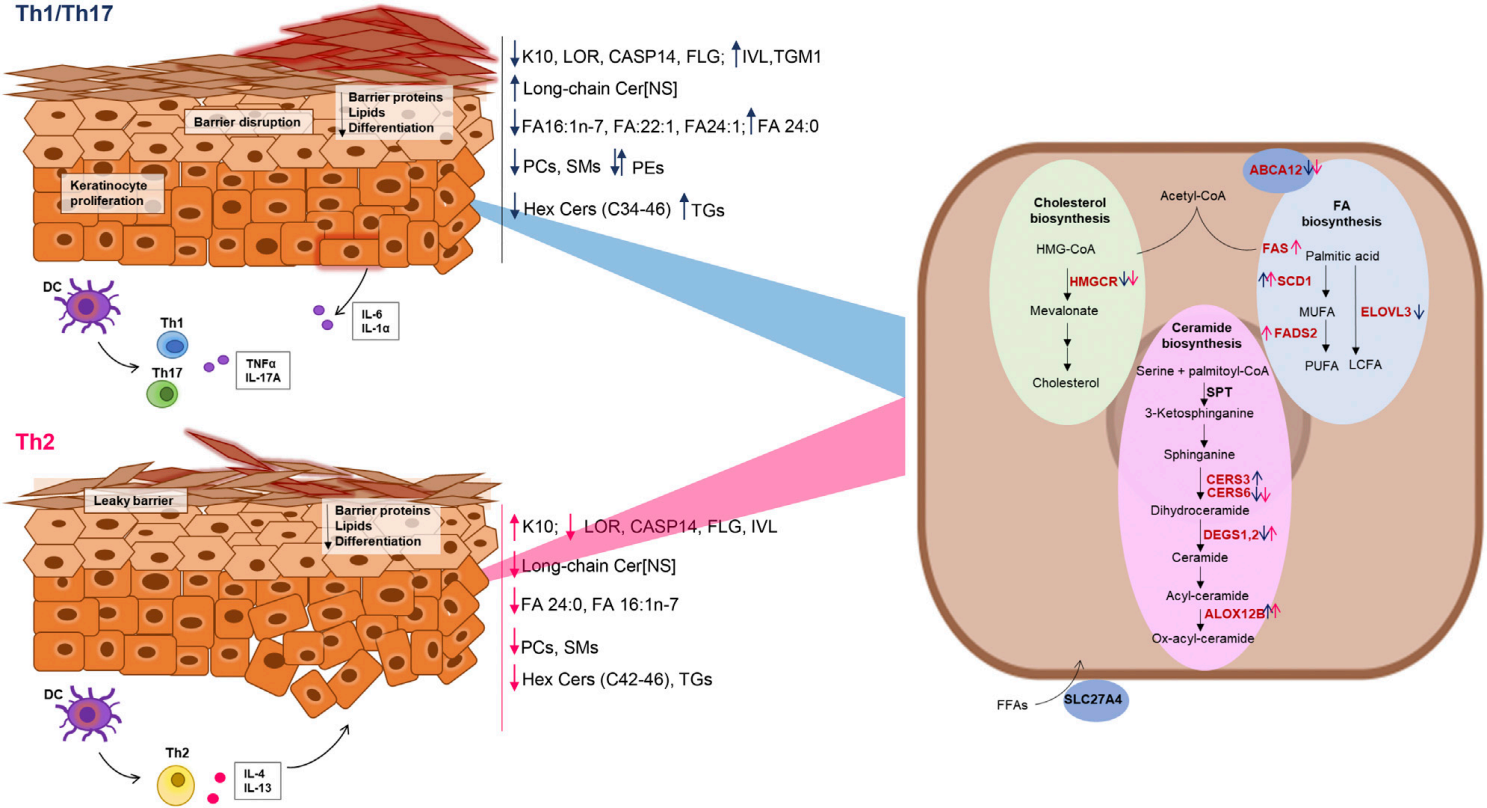
Schematic representation of the effects of Th1/Th17 and Th2 cytokines on protein and gene expression, and lipid modifications in differentiated keratinocytes. Blue and dark pink arrows indicate the effects of Th1/Th17 and Th2 cytokine mixtures respectively. The genes of the lipid pathways depicted in the keratinocyte cartoon on the right are in red or in black, respectively, when their expression was modulated or unmodulated by the Th1/Th17 or the Th2 cytokine signals.

To gain insights into the influence of the cytokines under investigation, we opted to integrate the findings from the 2D system with the lipidomic analyses conducted in 3D organotypic cultures. In contrast to conventional 2D cell culture, the reconstruction of 3D equivalents can mimic human epidermis in terms of layering, differentiation and barrier formation. The exposure of HEEs to mixtures of Th1 (TNF-α)/Th17 (IL-17A) plus IL-6 and IL-1α, or Th2 (IL-4, IL-13) cytokines during the last 5 days of the air-liquid interphase culture, induced dissimilar phenotypes. The HEEs showed a well multilayered and differentiated epidermis with the presence of the stratum corneum (Supplementary Figure S4A). Stimulation with psoriasis-associated cytokines affected epidermal morphology, resulting in loss of the stratum granulosum, thickening of the stratum corneum and parakeratosis (Supplementary Figure S4B). Instead, AD-associated cytokines induced signs of spongiosis, as evidenced by the presence of intercellular spaces between adjacent keratinocytes (Supplementary Figure S4C). The lipids were extracted from HEEs and analyzed with the same procedure applied to 2D cultures. HEEs cultures enabled the identification of a wide range of ceramides and a distinct group of hexosylceramides. Moreover, the list of analysed species was expanded to include the determination of TGs and DGs, in addition to FFAs, CHS, and phospholipids, resulting in a total of 300 targeted species. One-way analysis of variance (ANOVA) of the lipidomics data supported comparation among the groups (i.e., control, Th1/Th17 and Th2). The test retrieved 141 lipid species that showed statistically significant differences (Supplementary Table S5). Filtering out the species with FC < 2 versus the unchallenged samples, retrieved 64 lipid species. To explore relationships and correlations between lipids, the expression profiles of the 64 species were organized in a hierarchical clustering (Figure 5). The three columns represent the data for each experimental condition, expressed as the mean of six samples per group. The colour intensity indicates the logarithmically transformed abundance of each lipid species, ranging from dark blue (low abundance) to red (high abundance). The dendrogram supports the identification of shared behaviours and interrelationships among the lipids in the three conditions (Supplementary Table S6). The hierarchical clustering illustrates that the 12 most abundant entities in the 3D system (predominantly TGs and Cer [NS]34:1) are grouped together. These species are distinguished by an upregulation induced by Th1/Th17, whereas Th2 appears to exert a minimal influence. A second cluster of 13 species, i.e., TGs and ceramides of different subclasses, displays a parallel behavioural response to the two cytokines. Nevertheless, these species are distinguished by a relatively lower abundance. Two distinct groups are evident in the central region of the heatmap. A cluster of five species demonstrates a downregulation induced by both Th1/Th17 and Th2 cytokines, which includes palmitoleic acid (FA 16:1n-7). The second cluster encompasses 12 lipids, including members of the TG class and long-chain ceramides. This group exhibits a notable increase in response to Th1/Th17 cytokines and a mild decrease in response to Th2. In the lower region of the heatmap, which is characterised by the less abundant species in the 3D system, the dendrogram highlights a significant depletion of HexCers with chain length in the range 34–46 carbon atoms, induced by Th1/Th17. The depletion of HexCers observed with Th2 cytokines was statistically significant only for the species with chain length in the range 42–46 carbon atoms (Supplementary Figure S5). Furthermore, an additional group can be identified which corroborates the observed increase in TGs and ceramides, which is promoted by Th1/Th17. Lignoceric acid (FA 24:0) is a constituent of this group. All results of expression analyses and lipidomics were summarized in Figure 6.

## Discussion

Th1/Th17 and Th2 cytokines are primary contributors to the pathogenesis of psoriasis and AD, which exhibit deranged differentiation and lipid abnormalities in the epidermis. The differentiation program of keratinocytes is tightly intertwined with the generation of the hydrophobic EPB (Berdyshev et al., 2018; Pavel et al., 2022). The gradient of the calcium concentration in the epidermis regulates several intracellular events deploying the differentiation program leading to the functional EPB. The events of the abnormal epidermal differentiation characteristic of the Th1/Th17 and Th2 environments are not fully elucidated. Several models have been optimized to mimic *in vitro* the complex inflammatory milieus of AD or psoriasis using different cytokine mixtures (Ubago-Rodríguez et al., 2024; Alsabbagh, 2024; Quílez et al., 2024). The Th2 cytokines IL-4 and IL-13 are commonly used to model AD. For the psoriasis model, the most studied cytokines are TNF-α, IL-6, IL-1α, IL-17A, and IL-22 (Ubago-Rodríguez et al., 2024; Alsabbagh, 2024; Quílez et al., 2024). Some authors also evaluated the effects of the Th1-type IFN-γ (Chiricozzi et al., 2014; Nograles et al., 2008). In this study, we investigated the distinctive and shared responses to Th1/Th17 and Th2 cytokine mixtures on 2D cultures induced to differentiate, and organotypic models of epidermis using Ker-CT cells. The comparison of the responses to Th1/Th17 and Th2 cytokines provided matching results in terms of differentiation and lipid metabolism modulation in Ker-CT and human primary keratinocytes. In 2D cultures of Ker-CT, the calcium-induced protein expression of IVL, LOR, and FLG was tendentially decreased by both cytokine types, whereas K10 expression was significantly decreased and increased in keratinocytes treated with Th1/Th17 and Th2 cytokines, respectively. These results are in agreement with the decreased expression of K10 in psoriasis (Totsuka et al., 2017), in contrast to what observed in AD (Bovenschen et al., 2005). IVL expression is induced by IL-13, IL-17A, ET-1, TNF-α, and IFN-γ in both psoriatic and normal keratinocytes (Chen et al., 2013). In our study, opposite to the IVL protein levels, the *IVL* mRNA expression promoted by calcium was further elevated upon Th1/Th17 challenge. This divergent regulation may suggest the promotion of IVL removal consequent to Th1/Th17 cues (Zieba et al., 2017). Consistent to what observed for the LOR protein levels, both Th1/Th17 and Th2 cytokine mixtures decreased the *LOR* mRNA expression, as previously reported (Rabeony et al., 2014; Kim et al., 2008). The FLG protein expression was decreased by both cytokine types along a prominent downregulation of *CASP14*, which processes profilaggrin to FLG (Hoste et al., 2011). This result supports the detrimental effects of inflammatory cytokines exerted directly on the EPB (Hvid et al., 2011). TGM1 plays an essential role in skin barrier formation by cross-linking proteins in differentiated keratinocytes. In our model, Th1/Th17 and Th2 stimulation induced opposite effects on the *TGM1* mRNA expression promoted by high calcium, in agreement with its pronounced expression in psoriatic lesions (Surbek et al., 2023) and no significant changes in AD lesion (Liedén et al., 2012).

Both the formation and the integrity of the EPB are governed by the build-up of epidermal lipids, i.e., cholesterol, ceramides, and FFAs (Bhattacharya et al., 2019). In healthy SC, lipids are arranged predominantly in a dense lamellar formation (Danso et al., 2017). Several lipogenic pathways were regulated in the differentiated Ker-CT. The downregulation of *FAS, FADS2,* and *SCD1* genes and the upregulation of genes involved in the synthesis of cholesterol, long chain FFAs, and ceramides synthesis, and in the lipid transport, suggest differential effects at both early and late events of the lipogenic cascade during the differentiation process induced by high calcium.

The increase in HMGCR gene transcription observed in differentiated keratinocytes supports an increase in the synthesis of cholesterol and its derivatives, CEs and CHS, to preserve the integrity of the EPB (Elias et al., 2014). The elevation of the cholesterol levels occurring in the psoriatic epidermis (Nowowiejska et al., 2021) inhibits the expression of HMGCR through a feedback mechanism (Varshney et al., 2016; Jiang et al., 2010). Accordingly, we observed that both Th1/Th17 and Th2 environments reduced the transcription of the *HMGCR* gene. Channeling cholesterol excess in CEs is essential in cholesterol homeostasis. The elevation of the CEs concentration in differentiated keratinocytes was further increased in the Th1/Th17 environment. IFN-γ, IL-1, TNF-α, and LPS increase the CEs concentration in monocytes and macrophages, with a mechanism dependent on the NF-kB pathway (Luo et al., 2023). Elevation of the CHS concentration contributes to barrier dysfunction in AD (Li et al., 2016). Th2 cytokines exerted minimal effects on CHS accumulation in the used culturing conditions and concentrations, limiting the conclusions on the complex regulation of downstream cholesterol pathways (Li et al., 2016). Ceramides are lipid metabolites serving fundamental functions in the EPB homeostasis. In late stages of keratinocyte differentiation, ceramides undergo substantial changes resulting in a significant upregulation (Uchida and Park, 2021). The first step in the *de novo* synthesis of ceramides is catalyzed by the serine palmitoyltransferase (SPT) (Bhattacharya et al., 2019; Nowowiejska et al., 2021) which operates together with DEGS1 to form sphingosine. In mammalian cells, six enzymes of the ceramide synthase family (CERS1-6) catalyse binding of the FA moiety to the amino group of the sphinganine base to form ceramides with different chain lengths (Raichur, 2020). CERS3 is found mainly in the skin and its expression increases during keratinocyte differentiation (Cingolani et al., 2016). Alterations in both the profile and the metabolism of ceramides are implicated in inflammatory skin diseases (Luczaj et al., 2020; Pavel et al., 2022). Upon increase of calcium concentration, *de novo* synthesis of ceramides was considerably increased, as demonstrated by the elevation of both short and long chain ceramides. Th1/Th17 cytokines abrogated the formation of short chain ceramides, supporting the inhibition of the synthesis *de novo* observed in the psoriatic epidermis (Luczaj et al., 2020). Despite the parallel found in 2D system between the CERS gene expression and the corresponding NS ceramide produced upon challenge with Th1/Th17 or Th2 cytokines, other factors leading to the barrier formation are underrepresented. The use of HEEs enabled the exploration of a comprehensive panel of ceramides. Long chain-ceramides and acyl-ceramides were upregulated, while HexCers, where downregulated, by Th1/Th17 cytokines (Alessandrini et al., 2004). Th2 cytokines have a complex effect on ceramide profiles in differentiating keratinocytes (Berdyshev et al., 2018). Similarly to Th1/Th17, the Th2 cytokines resulted into decreased HexCers levels. In contrast, the Th2 cytokines caused a decrease in the acyl-ceramide levels, in keeping with the alterations of barrier lipids desribed in AD (Bhattacharya et al., 2019).

TG depletion in the epidermis is an initiating event and aggravating condition in AD. Effects of Th1/Th17 and Th2 cytokines on TG levels appear to occur in different directions in the HEEs model (Flori et al., 2024). FFAs play an important role in the function and homeostasis of the permeability barrier in the epidermis. The abundance of several MUFAs increased in differentiated keratinocytes. MUFA species, in particular palmitoleate (FA C16:1n-7) were downregulated by Th1/Th17 and Th2 cytokines in both 2D and 3D systems. Abnormalities in MUFAs levels could alter the normal skin architecture and disrupt lipid arrangement in lesional skin (Danso et al., 2017) by promoting a hexagonal packing conformation in contrast to the stable orthorhombic packing conformation (Mojumdar et al., 2014). Perturbation of the skin barrier has been shown to increase the mRNA levels of enzymes involved in the synthesis of FFAs (Bhattacharya et al., 2019). Alteration in the MUFA percentage is reported in AD (Bhattacharya et al., 2019; Danso et al., 2017). The evaluation of FFAs absolute amounts supports a significant depletion of FFAs upon Th2 challenge, in keeping with the lipid abnormalities found in the SC in AD (Cavallo et al., 2024). FFAs enter the keratinocytes via the fatty acid transport protein (FATP4), encoded by the *SLC27A4* gene (Schmuth et al., 2005). The decrease in SLC27A4 expression consequent to both cytokine types supports, at least in part, the decrease in FFA abundance (Khnykin et al., 2011). AD skin exhibits a distinctive reduction in very long-chain FAs across multiple lipid classes (Berdyshev et al., 2018). The ELOVL3 enzyme elongates SFAs from 16:0 to 22:0 (Zwara et al., 2021). ELOVL3 appears to be linked to the differentiation program, since its mRNA levels were increased significantly at later time of high calcium stimulation. Contrasting literature on ELOVLs gene and protein expression in *in vivo* and *in vitro* systems limits straightforward interpretations. In our experiments both Th1/Th17 and Th2 cytokine types interfere with calcium-promoted ELOVL3 expression, downmodulating its protein level. There is evidence that FFAs are toxic to living cells. In fact, normally, they are not stored in lamellar bodies in their free form, but principally as phospholipids, which do not degrade spontaneously. Phospholipids metabolism at the SG-SC interface results in the potential release of FFAs, which could affect proper barrier formation (Bhattacharya et al., 2019). Ker-CT cells cultured in high calcium concentration, showed a significant upregulation in all phosholipid classes, with the exception of some PE members. There is evidence of higher levels of PEs in lesional and lesion-free atopic epidermis compared to healthy controls (Schäfer and Kragballe, 1991). However, our results point out at specific effects on PE members, as some PEs were upregulated and some PEs downregulated. PCs are prominent components of the plasma membrane. We observed a significant decrease in PCs with multiple unsaturations with Th2-cytokines, suggesting that Th2-signalling may affect the barrier architecture through disruption of the PCs homeostasis. Psoriasis skin exhibits elevated levels of total PCs and a PC subset presenting both ester and ether bound side chains, which can be attributed to abnormal epidermal hyperproliferation (Ilves et al., 2022; Pietrzak et al., 2010). Differentiated keratinocytes treated with Th1/Th17 cytokines in submerged conditions showed a significant reduction in PCs. In HEEs, PC 28:0 increased 2-fold upon Th1/Th17 challenge. These discrepancies are likely due to the *in vitro* model lacking the contributing factors to the overall changes in PCs occurring *in vivo* skin. Together with PCs and PEs, SMs are major components of the cell. The significant induction of SMs by high calcium was contrasted by Th1/Th17 cytokines, suggesting that lipid changes may occur before cornification (Proksch et al., 2008). SMs together with glucosylceramides are immediate precursors of ceramide in the SC. Likely, the submerged culturing conditions are unfavourable for the formation or accumulation of a complete spectrum of sphingolipids, as demonstrated by the very low abundance of glucosylceramides that limited their unequivocal identification in 2D cultures. In contrast, the glucosylceramides could be determined in HEEs, although these lipids were accounted among the least abundant ones. Recently, the analysis of the lipidome is gaining importance as a tool to predict the severity and the progression of inflammatory skin diseases (Nowowiejska et al., 2023). In this study, we could observe the specific and shared apparent effects that cytokines of the Th1/Th17 and Th2 types exerted on the lipid profiles of keratinocytes induced to differentiate with high calcium and in HEEs, confirming the impact of cytokines on the EPB functions. Interestingly, the activation of several lipid pathways occurred at an extent that matched the abundance of the corresponding lipid metabolite. The wide range of conditions (e.g., experimental models, cytokine cocktails, doses and times of treatment) adopted for the investigation of the immune environment effects on epidermal homeostasis limits straightforward comparison of the experimental data. Investigations performed in diverse experimental systems, emphasize findings dependent and independent on the variation of both the pro-differentiating and immune environments. The present study contributes to the current knowledge on the lipid metabolism targeted by inflammatory cytokines, useful in the definition of strategies aimed at ameliorating intrinsic epidermal defects and at restoring proper skin barrier function.

## Data Availability

The original contributions presented in the study are included in the article/Supplementary Material, further inquiries can be directed to the corresponding author.

## Glossary

AD: atopic dermatitis
ABCA12: ATP Binding Cassette Subfamily A Member 12
ALOX12B: arachidonate 12-lipoxygenase
ANOVA: one-way analysis of variance
BSTFA: N,O-bis(trimethyl-silyl)-trifluoroacetamide
**↑Ca** ^**2+**^: high calcium
CA2: carbonic anhydrase 2
CASP14: caspase 14
CCL26: C-C motif chemokine ligand 26
CHS: cholesterol sulfate
DEGS1: sphingolipid 1 delta (4)-desaturase
DEGS2: sphingolipid 2 delta (4)-desaturase
CERS1-6: ceramide synthase family
EPB: epidermal permeability barrier
ELOVLs: fatty acids elongases
FADS: fatty acid desaturases
FAS: fatty acid synthase
FATP4: fatty acid transport protein
FFAs: free fatty acids
FLG: filaggrin
FC: fold change
GAPDH: glyceraldehyde-3-phosphate dehydrogenase
GCMS: gas chromatography-mass spectrometry
HEE: human epidermal equivalent
HMGCR: 3-hydroxy-3-methylglutaryl-CoA reductase
HPLC: high performance liquid chromatography
HRMS: high resolution mass spectrometry
HILIC: hydrophilic interaction liquid chromatography
IL: interleukin
IVL: involucrin
JAK: Janus kinase
K10: keratin 10
LCMS: liquid chromatography-mass spectrometry
LOR: loricrin
LPCs: lysophosphatidylcholines
MUFAs: monounsaturated fatty acids
PCs: phosphatidylcholines
PEs: phosphatidylethanolamines
PPARγ: peroxisome proliferator activated receptor gamma
Q-TOF: quadrupole-time of flight
RP-HPLC: reversed phase-HPLC
SC: stratum corneum
SCD1: stearoyl-CoA desaturase
SFAs: saturated fatty acids
SG: stratum granulosum
SLC27A4: solute carrier family 27 member 4
SMs: sphingomyelins
SPT: serine palmitoyltransferase
STAT: signal transducer and activator of transcription
Th1/Th17: T-helper1/T-helper17
Th2: T-helper2
TGM1: Transglutaminase 1
TNF-α: Tumor necrosis factor alpha
TCMS: trimethylchlorosilane.

## References

[B1] Akiyama M. (2014). The roles of ABCA12 in epidermal lipid barrier formation and keratinocyte differentiation. Biochim. Biophys. Acta 1841, 435–440. 10.1016/j.bbalip.2013.08.009 23954554

[B2] Alessandrini F. Pfister S. Kremmer E. Gerber J. Ring J. Behrendt H. (2004). Alterations of glucosylceramide-beta-glucosidase levels in the skin of patients with psoriasis vulgaris. J. Invest Dermatol 123, 1030–1036. 10.1111/j.0022-202X.2004.23469.x 15610510

[B3] Alsabbagh M. M. (2024). Cytokines in psoriasis: from pathogenesis to targeted therapy. Hum. Immunol. 85, 110814. 10.1016/j.humimm.2024.110814 38768527

[B4] Beckert B. Panico F. Pollmann R. Eming R. Banning A. Tikkanen R. (2019). Immortalized human hTert/KER-CT keratinocytes a model system for research on desmosomal adhesion and pathogenesis of pemphigus vulgaris. Int. J. Mol. Sci. 20, 3113. 10.3390/ijms20133113 31247885 PMC6651391

[B5] Berdyshev E. Goleva E. Bronova I. Dyjack N. Rios C. Jung J. (2018). Lipid abnormalities in atopic skin are driven by type 2 cytokines. JCI Insight 3, e98006. 10.1172/jci.insight.98006 29467325 PMC5916244

[B6] Bhattacharya N. Sato W. J. Kelly A. Ganguli-Indra G. Indra A. K. (2019). Epidermal lipids: key mediators of atopic dermatitis pathogenesis. Trends Mol. Med. 25, 551–562. 10.1016/j.molmed.2019.04.001 31054869 PMC6698381

[B7] Bieber T. (2020). Interleukin-13: targeting an underestimated cytokine in atopic dermatitis. Allergy 75, 54–62. 10.1111/all.13954 31230370

[B8] Bikle D. D. Xie Z. Tu C. (2012). Calcium regulation of keratinocyte differentiation. Expert Rev. Endocrinol. Metab. 7, 461–472. 10.1586/eem.12.34 23144648 PMC3491811

[B9] Borowiec A. Delcourt P. Dewailly E. Bidaux G. (2013). Optimal differentiation of *in vitro* keratinocytes requires multifactorial external control. PLoS One 8, e77507. 10.1371/journal.pone.0077507 24116231 PMC3792032

[B10] Bovenschen H. J. Seyger M. M. B. Van de Kerkhof P. C. M. (2005). Plaque psoriasis vs. atopic dermatitis and lichen planus: a comparison for lesional T-cell subsets, epidermal proliferation and differentiation. Br. J. Dermatol 153, 72–78. 10.1111/j.1365-2133.2005.06538.x 16029329

[B11] Cavallo A. Camera E. Bottillo G. Maiellaro M. Truglio M. Marini F. (2024). Biosignatures of defective sebaceous gland activity in sebum‐rich and sebum‐poor skin areas in adult atopic dermatitis. Exp. Dermatol. 33, e15066. 10.1111/exd.15066 38532571

[B12] Chen J. Man X. Li W. Zhou J. Landeck L. Cai S. (2013). Regulation of involucrin in psoriatic epidermal keratinocytes: the roles of ERK1/2 and GSK-3β. Cell Biochem. Biophys. 66, 523–528. 10.1007/s12013-012-9499-y 23283814

[B13] Chen W. Chen S. Hsu S. Lin Y. Shih C. Huang C. (2022). Annoying psoriasis and atopic dermatitis: a narrative review. Int. J. Mol. Sci. 23, 4898. 10.3390/ijms23094898 35563285 PMC9104570

[B14] Chen W. Xu J. Wu Y. Liang B. Yan M. Sun C. (2023). The potential role and mechanism of circRNA/miRNA axis in cholesterol synthesis. Int. J. Biol. Sci. 19, 2879–2896. 10.7150/ijbs.84994 37324939 PMC10266072

[B15] Chieosilapatham P. Kiatsurayanon C. Umehara Y. Trujillo‐Paez J. V. Peng G. Yue H. (2021). Keratinocytes: innate immune cells in atopic dermatitis. Clin. Exp. Immunol. 204, 296–309. 10.1111/cei.13575 33460469 PMC8119845

[B16] Chiricozzi A. Nograles K. E. Johnson-Huang L. M. Fuentes-Duculan J. Cardinale I. Bonifacio K. M. (2014). IL-17 induces an expanded range of downstream genes in reconstituted human epidermis model. PLOS ONE 9, e90284. 10.1371/journal.pone.0090284 24587313 PMC3938679

[B17] Cingolani F. Futerman A. H. Casas J. (2016). Ceramide synthases in biomedical research. Chem. Phys. Lipids 197, 25–32. 10.1016/j.chemphyslip.2015.07.026 26248326

[B18] Danso M. Boiten W. van Drongelen V. Gmelig Meijling K. Gooris G. El Ghalbzouri A. (2017). Altered expression of epidermal lipid bio-synthesis enzymes in atopic dermatitis skin is accompanied by changes in stratum corneum lipid composition. J. Dermatol Sci. 88, 57–66. 10.1016/j.jdermsci.2017.05.005 28571749

[B19] Elias P. M. Williams M. L. Choi E. Feingold K. R. (2014). Role of cholesterol sulfate in epidermal structure and function: lessons from X-linked ichthyosis. Biochim. Biophys. Acta 1841, 353–361. 10.1016/j.bbalip.2013.11.009 24291327 PMC3966299

[B20] Flori E. Cavallo A. Mosca S. Kovacs D. Cota C. Zaccarini M. (2024). JAK/STAT inhibition normalizes lipid composition in 3D human epidermal equivalents challenged with Th2 cytokines. Cells 13, 760. 10.3390/cells13090760 38727296 PMC11083560

[B21] Hoste E. Kemperman P. Devos M. Denecker G. Kezic S. Yau N. (2011). Caspase-14 is required for filaggrin degradation to natural moisturizing factors in the skin. J. Invest Dermatol 131, 2233–2241. 10.1038/jid.2011.153 21654840

[B22] Huang I.-Chung W. Wu P. Chen C. (2022). JAK-STAT signaling pathway in the pathogenesis of atopic dermatitis: an updated review. Front. Immunol. 13, 1068260. 10.3389/fimmu.2022.1068260 36569854 PMC9773077

[B23] Hvid M. Johansen C. Deleuran B. Kemp K. Deleuran M. Vestergaard C. (2011). Regulation of caspase 14 expression in keratinocytes by inflammatory cytokines--a possible link between reduced skin barrier function and inflammation? Exp. Dermatol 20, 633–636. 10.1111/j.1600-0625.2011.01280.x 21539619

[B24] Ilves L. Ottas A. Kaldvee B. Abram K. Soomets U. Zilmer M. (2022). Metabolomic differences between the skin and blood sera of atopic dermatitis and psoriasis. Int. J. Mol. Sci. 23, 13001. 10.3390/ijms232113001 36361789 PMC9658722

[B25] Ishikawa J. Narita H. Kondo N. Hotta M. Takagi Y. Masukawa Y. (2010). Changes in the ceramide profile of atopic dermatitis patients. J. Invest Dermatol 130, 2511–2514. 10.1038/jid.2010.161 20574438

[B26] Ito S. Ishikawa J. Naoe A. Yoshida H. Hachiya A. Fujimura T. (2017). Ceramide synthase 4 is highly expressed in involved skin of patients with atopic dermatitis. J. Eur. Acad. Dermatol Venereol. 31, 135–141. 10.1111/jdv.13777 27358008

[B27] Jiang Y. Tsoi L. C. Billi A. C. Ward N. L. Harms P. W. Zeng C. (2020). Cytokinocytes: the diverse contribution of keratinocytes to immune responses in skin. JCI insight 5, e142067. 10.1172/jci.insight.142067 33055429 PMC7605526

[B28] Jiang Y. J. Lu B. Tarling E. J. Kim P. Man M. Crumrine D. (2010). Regulation of ABCG1 expression in human keratinocytes and murine epidermis. J. Lipid Res. 51, 3185–3195. 10.1194/jlr.M006445 20675829 PMC2952559

[B29] Kendall A. C. Nicolaou A. (2022). Topical application of lipids to correct abnormalities in the epidermal lipid barrier. Br. J. Dermatol 186, 764–765. 10.1111/bjd.21294 35501940 PMC9321633

[B30] Khnykin D. Miner J. H. Jahnsen F. (2011). Role of fatty acid transporters in epidermis: implications for health and disease. Dermatoendocrinol 3, 53–61. 10.4161/derm.3.2.14816 21695012 PMC3117002

[B31] Kim B. E. Leung D. Y. M. Boguniewicz M. Howell M. D. (2008). Loricrin and involucrin expression is down-regulated by Th2 cytokines through STAT-6. Clin. Immunol. 126, 332–337. 10.1016/j.clim.2007.11.006 18166499 PMC2275206

[B32] Kok J. M. L. Dowd G. C. Cabral J. D. Wise L. M. (2023). Macrocystis pyrifera lipids reduce cytokine-induced pro-inflammatory signalling and barrier dysfunction in human keratinocyte models. Int. J. Mol. Sci. 24, 16383. 10.3390/ijms242216383 38003573 PMC10671590

[B33] Kovacs D. Maresca V. Flori E. Mastrofrancesco A. Picardo M. Cardinali G. (2020). Bovine colostrum induces the differentiation of human primary keratinocytes. FASEB J. 34, 6302–6321. 10.1096/fj.201900103RRR 32157742

[B34] Kuwatsuka S. Koike Y. Kuwatsuka Y. Yamaoka T. Murota H. (2021). Claudin-7 in keratinocytes is downregulated by the inhibition of HMG-CoA reductase and is highly expressed in the stratum granulosum of the psoriatic epidermis. J. Dermatol Sci. 104, 132–137. 10.1016/j.jdermsci.2021.10.002 34763991

[B35] Lamb R. Ambler C. A. (2013). Keratinocytes propagated in serum-free, feeder-free culture conditions fail to form stratified epidermis in a reconstituted skin model. PLOS ONE 8, e52494. 10.1371/journal.pone.0052494 23326335 PMC3543440

[B36] Li S. Ganguli-Indra G. Indra A. K. (2016). Lipidomic analysis of epidermal lipids: a tool to predict progression of inflammatory skin disease in humans. Expert Rev. Proteomics 13, 451–456. 10.1080/14789450.2016.1177462 27121756 PMC4939172

[B37] Liedén A. Winge M. C. G. Sääf A. Kockum I. Ekelund E. Rodriguez E. (2012). Genetic variation in the epidermal transglutaminase genes is not associated with atopic dermatitis. PLoS One 7, e49694. 10.1371/journal.pone.0049694 23189155 PMC3506648

[B38] Luczaj W. Wronski A. Domingues P. Domingues M. R. Skrzydlewska E. (2020). Lipidomic analysis reveals specific differences between fibroblast and keratinocyte ceramide profile of patients with psoriasis vulgaris. Molecules 25, 630. 10.3390/molecules25030630 32023992 PMC7037443

[B39] Ludovici M. Kozul N. Materazzi S. Risoluti R. Picardo M. Camera E. (2018). Influence of the sebaceous gland density on the stratum corneum lipidome. Sci. Rep. 8, 11500. 10.1038/s41598-018-29742-7 30065281 PMC6068117

[B40] Luo L. Guo Y. Chen L. Zhu J. Li C. (2023). Crosstalk between cholesterol metabolism and psoriatic inflammation. Front. Immunol. 14, 1124786. 10.3389/fimmu.2023.1124786 37234169 PMC10206135

[B41] Maiellaro M. Bottillo G. Cavallo A. Camera E. (2024). Comparison between ammonium formate and ammonium fluoride in the analysis of stratum corneum lipids by reversed phase chromatography coupled with high resolution mass spectrometry. Sci. Rep. 14, 40. 10.1038/s41598-023-50051-1 38167931 PMC10762128

[B42] Meyer J. M. Vávrová K. Radner F. P. W. Schneider H. Dick A. Mauro T. M. (2023). ALOX12B and PNPLA1 have distinct roles in epidermal lipid lamellar organization. J. Invest Dermatol 143, 332–335.e4. 10.1016/j.jid.2022.07.029 36116510

[B43] Mojumdar E. H. Helder R. W. Gooris G. S. Bouwstra J. A. (2014). Monounsaturated fatty acids reduce the barrier of stratum corneum lipid membranes by enhancing the formation of a hexagonal lateral packing. Langmuir 30, 6534–6543. 10.1021/la500972w 24818519

[B44] Nograles K. E. Zaba L. C. Guttman-Yassky E. Fuentes-Duculan J. Suárez-Fariñas M. Cardinale I. (2008). Th17 cytokines interleukin (IL)-17 and IL-22 modulate distinct inflammatory and keratinocyte-response pathways. Br. J. Dermatol 159, 1092–1102. 10.1111/j.1365-2133.2008.08769.x 18684158 PMC2724264

[B45] Nowowiejska J. Baran A. Flisiak I. (2021). Aberrations in lipid expression and metabolism in psoriasis. Int. J. Mol. Sci. 22, 6561. 10.3390/ijms22126561 34207318 PMC8234564

[B46] Nowowiejska J. Baran A. Flisiak I. (2023). Lipid alterations and metabolism disturbances in selected inflammatory skin diseases. Int. J. Mol. Sci. 24, 7053. 10.3390/ijms24087053 37108216 PMC10138531

[B47] Ota A. Morita H. Naganuma T. Miyamoto M. Jojima K. Nojiri K. (2023). Bifunctional DEGS2 has higher hydroxylase activity toward substrates with very-long-chain fatty acids in the production of phytosphingosine ceramides. J. Biol. Chem. 299, 104603. 10.1016/j.jbc.2023.104603 36907437 PMC10140171

[B48] Park H. Haynes C. A. Nairn A. V. Kulik M. Dalton S. Moremen K. (2010). Transcript profiling and lipidomic analysis of ceramide subspecies in mouse embryonic stem cells and embryoid bodies. J. Lipid Res. 51, 480–489. 10.1194/jlr.M000984 19786568 PMC2817578

[B49] Pavel P. Blunder S. Moosbrugger-Martinz V. Elias P. M. Dubrac S. (2022). Atopic dermatitis: the fate of the fat. Int. J. Mol. Sci. 23, 2121. 10.3390/ijms23042121 35216234 PMC8880331

[B50] Piasek A. M. Levkovych I. Musolf P. Chmielewska H. Ścieżyńska A. Sobiepanek A. (2023). Building up skin models for numerous applications - from two-dimensional (2D) monoculture to three-dimensional (3D) multiculture. J. Vis. Exp. 10.3791/65773 37930006

[B51] Pietrzak A. Michalak-Stoma A. Chodorowska G. Szepietowski J. C. (2010). Lipid disturbances in psoriasis: an update. Mediat. Inflamm. 2010, 535612. 10.1155/2010/535612 PMC2914266 20706605

[B52] Planas-Serra L. Launay N. Goicoechea L. Heron B. Jou C. Juliá-Palacios N. (2023). Sphingolipid desaturase DEGS1 is essential for mitochondria-associated membrane integrity. J. Clin. Invest 133, e162957. 10.1172/JCI162957 36951944 PMC10178845

[B53] Proksch E. Brandner J. M. Jensen J. (2008). The skin: an indispensable barrier. Exp. Dermatol 17, 1063–1072. 10.1111/j.1600-0625.2008.00786.x 19043850

[B54] Quílez C. Bebiano L. B. Jones E. Maver U. Meesters L. Parzymies P. (2024). Targeting the complexity of *in vitro* skin models: a review of cutting-edge developments. J. Investigative Dermatology 144, 2650–2670. 10.1016/j.jid.2024.04.032 39127929

[B55] Rabeony H. Petit-Paris I. Garnier J. Barrault C. Pedretti N. Guilloteau K. (2014). Inhibition of keratinocyte differentiation by the synergistic effect of IL-17A, IL-22, IL-1α, TNFα and oncostatin M. PLoS One 9, e101937. 10.1371/journal.pone.0101937 25010647 PMC4092099

[B56] Raichur S. (2020). Ceramide synthases are attractive drug targets for treating metabolic diseases. Front. Endocrinol. (Lausanne) 11, 483. 10.3389/fendo.2020.00483 32849276 PMC7403459

[B57] Reijnders C. M. A. van Lier A. Roffel S. Kramer D. Scheper R. J. Gibbs S. (2015). Development of a full-thickness human skin equivalent *in vitro* model derived from TERT-immortalized keratinocytes and fibroblasts. Tissue Eng. Part A 21, 2448–2459. 10.1089/ten.TEA.2015.0139 26135533 PMC4554934

[B58] Schäfer L. Kragballe K. (1991). Abnormalities in epidermal lipid metabolism in patients with atopic dermatitis. J. Investigative Dermatology 96, 10–15. 10.1111/1523-1747.ep12514648 1987285

[B59] Schmuth M. Ortegon A. M. Mao-Qiang M. Elias P. M. Feingold K. R. Stahl A. (2005). Differential expression of fatty acid transport proteins in epidermis and skin appendages. J. Invest Dermatol. 125, 1174–1181. 10.1111/j.0022-202X.2005.23934.x 16354187

[B60] Smits J. P. H. Niehues H. Rikken G. van Vlijmen-Willems I. M. J. J. van de Zande G. W. H. J. F. Zeeuwen P. L. J. M. (2017). Immortalized N/TERT keratinocytes as an alternative cell source in 3D human epidermal models. Sci. Rep. 7, 11838. 10.1038/s41598-017-12041-y 28928444 PMC5605545

[B61] Surbek M. Van de Steene T. Sachslehner A. P. Golabi B. Griss J. Eyckerman S. (2023). Cornification of keratinocytes is associated with differential changes in the catalytic activity and the immunoreactivity of transglutaminase-1. Sci. Rep. 13, 21550. 10.1038/s41598-023-48856-1 38057394 PMC10700374

[B62] Teshima H. Endo M. Furuyama Y. Takama H. Akiyama M. Tsuji T. (2023). Involvement of hypoxia‐inducible factor activity in inevitable air‐exposure treatment upon differentiation in a three‐dimensional keratinocyte culture. FEBS J. 290, 2049–2063. 10.1111/febs.16707 36549886

[B63] Totsuka A. Omori-Miyake M. Kawashima M. Yagi J. Tsunemi Y. (2017). Expression of keratin 1, keratin 10, desmoglein 1 and desmocollin 1 in the epidermis: possible downregulation by interleukin-4 and interleukin-13 in atopic dermatitis. Eur. J. Dermatol 27, 247–253. 10.1684/ejd.2017.2985 28524044

[B64] Ubago-Rodríguez A. Quiñones-Vico M. I. Sánchez-Díaz M. Sanabria-de la Torre R. Sierra-Sánchez Á. Montero-Vílchez T. (2024). Challenges in psoriasis research: a systematic review of preclinical models. Dermatology 240, 620–652. 10.1159/000538993 38857576

[B65] Uchida Y. Park K. (2021). Ceramides in skin health and disease: an update. Am. J. Clin. Dermatol. 22, 853–866. 10.1007/s40257-021-00619-2 34283373

[B66] Varshney P. Narasimhan A. Mittal S. Malik G. Sardana K. Saini N. (2016). Transcriptome profiling unveils the role of cholesterol in IL-17A signaling in psoriasis. Sci. Rep. 6, 19295. 10.1038/srep19295 26781963 PMC4726068

[B67] Weidinger S. Beck L. A. Bieber T. Kabashima K. Irvine A. D. (2018). Atopic dermatitis. Nat. Rev. Dis. Prim. 4, 1. 10.1038/s41572-018-0001-z 29930242

[B68] Xie Z. Singleton P. A. Bourguignon L. Y. W. Bikle D. D. (2005). Calcium-induced human keratinocyte differentiation requires src- and fyn-mediated phosphatidylinositol 3-kinase-dependent activation of phospholipase C-gamma1. Mol. Biol. Cell 16, 3236–3246. 10.1091/mbc.E05-02-0109 15872086 PMC1165407

[B69] Zieba B. A. Henry L. Lacroix M. Jemaà M. Lavabre-Bertrand T. Meunier L. (2017). The proteasome maturation protein POMP increases proteasome assembly and activity in psoriatic lesional skin. J. Dermatol Sci. 88, 10–19. 10.1016/j.jdermsci.2017.04.009 28728908

[B70] Zwara A. Wertheim-Tysarowska K. Mika A. (2021). Alterations of ultra long-chain fatty acids in hereditary skin diseases—review article. Front. Med. (Lausanne) 8, 730855. 10.3389/fmed.2021.730855 34497816 PMC8420999

