# Supplementary material for: Effects of Th1/Th17 and Th2 cytokines on lipid metabolism in differentiated keratinocytes
Source: Front Physiol. 2025 Feb 19;16:1387128. doi: 10.3389/fphys.2025.1387128 (PMC11880217; doi:10.3389/fphys.2025.1387128)
Supplement: Supplementary file 1 [file DataSheet1.zip › Supplementary Data Sheet/1 Supplementary Material S1.pdf]

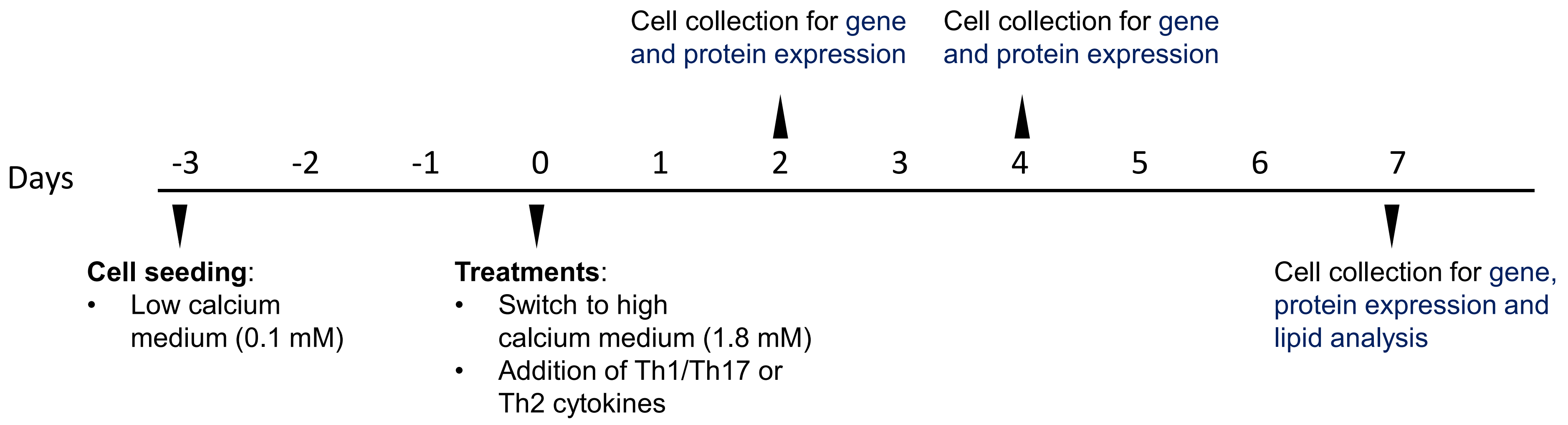

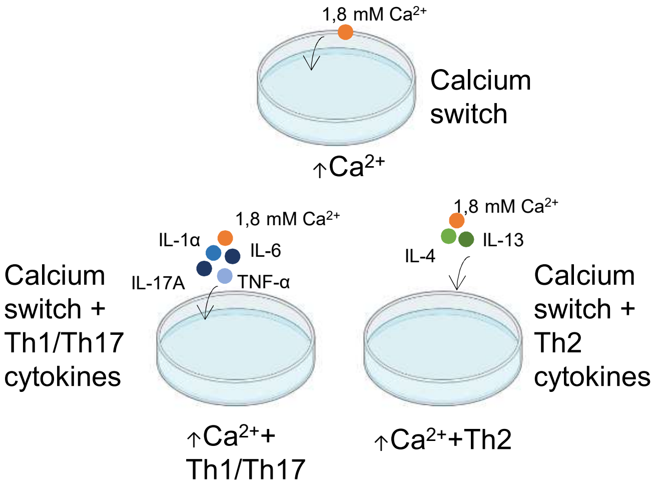

Samples	Abbreviation	Treatment	Description
Control	Ctr	0.1 mM Ca^{2+}	Actively proliferating keratinocytes maintained in complete culture medium (with growth factors) with low calcium concentration
High calcium	↑Ca^{2+}	1.8 mM Ca^{2+}	Keratinocytes induced to differentiation cultured in complete medium with high calcium concentration
High calcium combined to Th1/Th17	↑Ca^{2+} + Th1/Th17	1.8 mM Ca^{2+} + TNFα, IL-6, IL-1α, IL-17A	*In vitro* psoriasis model. Keratinocytes in complete medium and high calcium stimulated with a mixture of pro-inflammatory Th1/Th17 cytokines
High calcium combined to Th2	↑Ca^{2+} + Th2	1.8 mM Ca^{2+} + IL-4, IL-13	*In vitro* atopic dermatitis model. Keratinocytes in complete medium and high calcium stimulated with a mixture of pro-inflammatory Th2 cytokines

EXPERIMENTAL DESIGN
