# Supplementary material for: Effects of Th1/Th17 and Th2 cytokines on lipid metabolism in differentiated keratinocytes
Source: Front Physiol. 2025 Feb 19;16:1387128. doi: 10.3389/fphys.2025.1387128 (PMC11880217; doi:10.3389/fphys.2025.1387128)
Supplement: Supplementary file 1 [file DataSheet1.zip › Supplementary Data Sheet/2 Supplementary Figures S1-S5.pdf]

Supplementary Figures

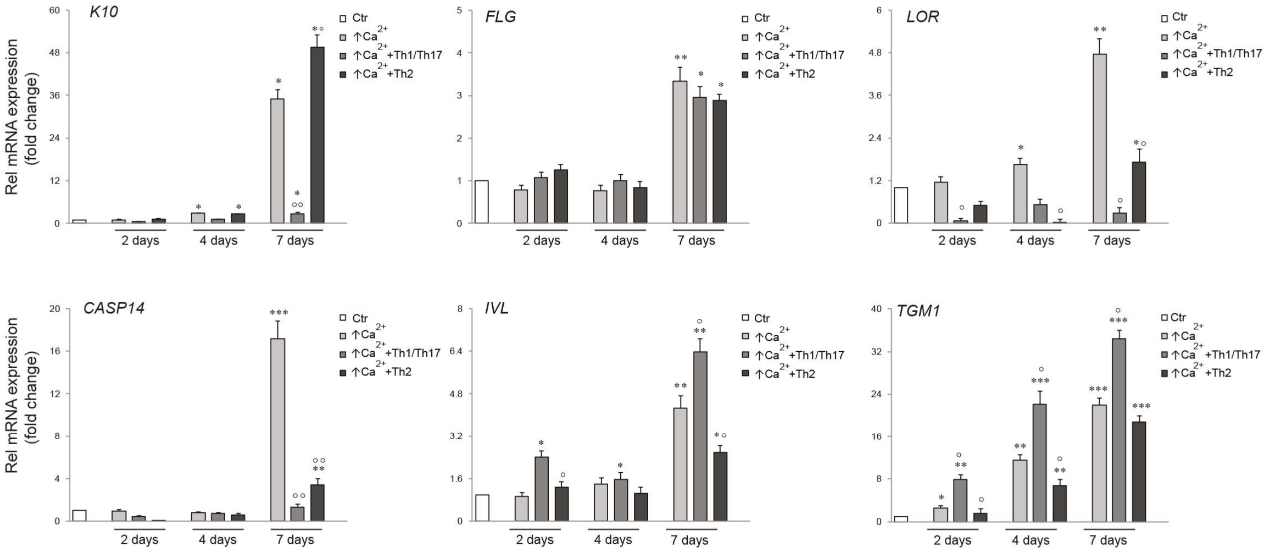

Supplementary Figure S1

Supplementary Figure S1. Quantitative real time PCR analysis of genes involved in keratinocyte differentiation in Ker-CT cells treated with ↑Ca^{2+}, ↑Ca^{2+}+Th1/Th17 and ↑Ca^{2+}+Th2 for 2, 4 and 7 days. All mRNA values were normalized against the expression of GAPDH and were expressed as relative to low calcium control (Ctr). Data represent the mean ± SD of three independent experiments (significance vs low calcium control or vs stimulated cells with ↑Ca^{2+} are marked with * and °, respectively; *$p < 0.05$, **$p < 0.01$ and ***$p < 0.001$ vs low calcium cells; °$p < 0.05$, °°$p < 0.01$ vs differentiated cells). Results are expressed as fold change relative to the value of cells grown in low calcium, which was set as 1 by definition.

(A)

	7 days		
**Differentiation**	**↑Ca^{2+}**	**↑Ca^{2+} + Th1/Th17**	**↑Ca^{2+} +Th2**
K10	59.8±2.87	1.02±0.02	73.4±5.80
FLG	32.2±1.96	1.30±0.07	10.7±0.41
LOR	96.5±5.69	1.79±0.07	7.43±0.47
IVL	27.9±1.11	30.1±1.98	13.79±0.62
CASP14	11.0±0.55	0.75±0.02	6.06±0.27
**Lipid metabolism**	**↑Ca^{2+}**	**↑Ca^{2+} + Th1/Th17**	**↑Ca^{2+} +Th2**
CERS4	10.99±0.94	6.29±0.56	8.63±0.24
CERS6	5.01±0.41	1.95±0.16	4.89±0.15
FAS	0.79±0.06	0.75±0.03	1.20±0.06
FADS2	0.15±0.01	0.47±0.03	0.57±0.01
HMGCR	4.71±0.24	2.29±0.09	3.73±0.42
DEGS1	11.6±0.87	2.29±0.09	11.3±0.43
DEGS2	50.4±4.49	36.0±2.63	68.3±1.99
ABCA12	35.2±2.65	12.8±1.06	25.1±1.46
SLC27A4	7.39±0.55	3.41±0.19	5.90±0.35
ELOVL3	18.4±1.13	0.55±0.06	7.53±0.58

(B)

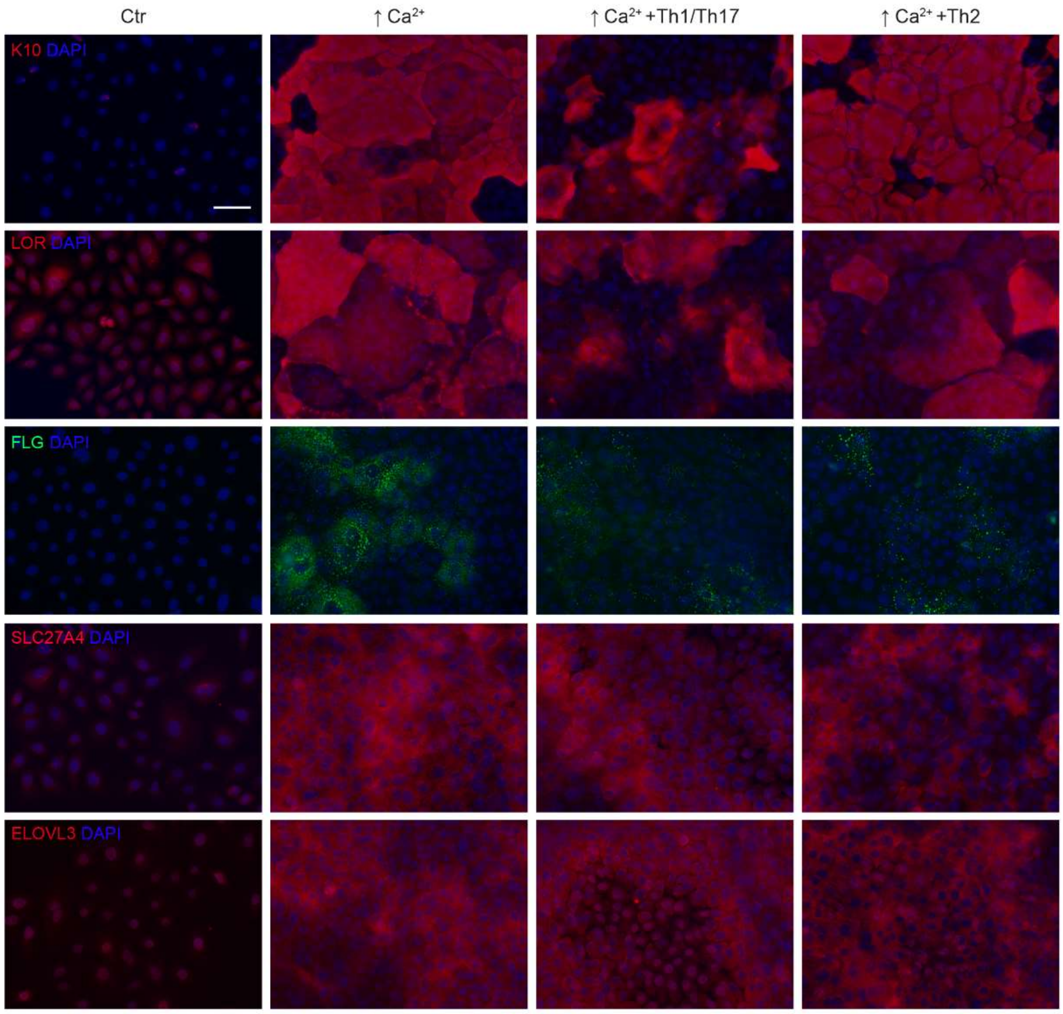

Supplementary Figure S2

Supplementary Figure S2.

(A) Fold changes (mean ± SD) of mRNA expression of genes involved in keratinocyte differentiation and lipid metabolism in human primary keratinocytes treated with high calcium (↑Ca^{2+}), high calcium with Th1/Th17 cytokines (↑Ca^{2+} + Th1/Th17) and high calcium with Th2 cytokines (↑Ca^{2+} + Th2) for 7 days compared to low calcium control. (B) Immunofluorescence analysis of K10, LOR, FLG, SLC27A4, and ELOVL3 following stimulation with Th1/Th17 or Th2 cytokine types in high calcium condition. Nuclei are counterstained in DAPI. Scale bar: 50 μm.

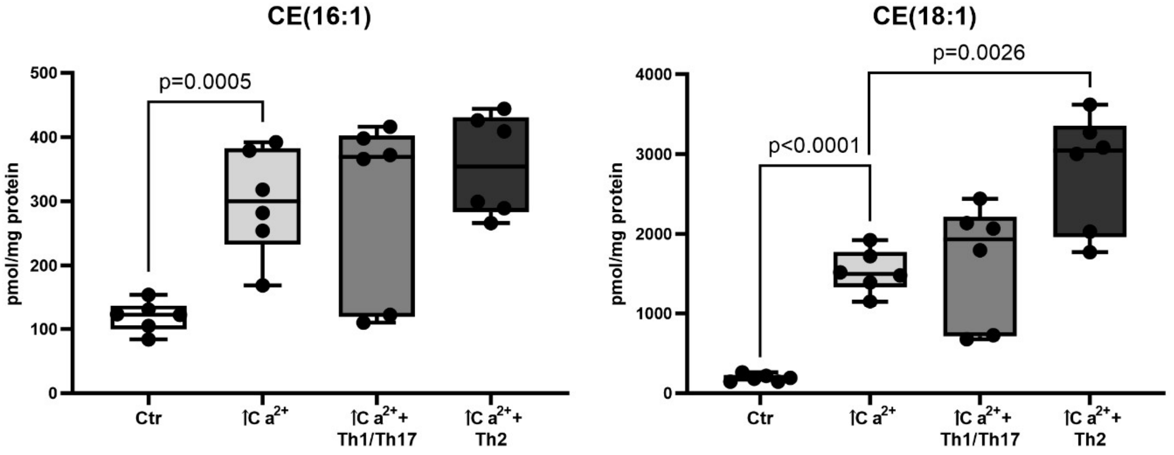

Supplementary Figure S3

Supplementary Figure S3. Concentration of CE(16:1) and CE(18:1) quantified in lipid extracts in low calcium Ker-CT cells (Ctr) and treated with ↑Ca^{2+}, ↑Ca^{2+}+Th1/Th17, ↑Ca^{2+}+Th2 against the deuterated cholesterol ester.

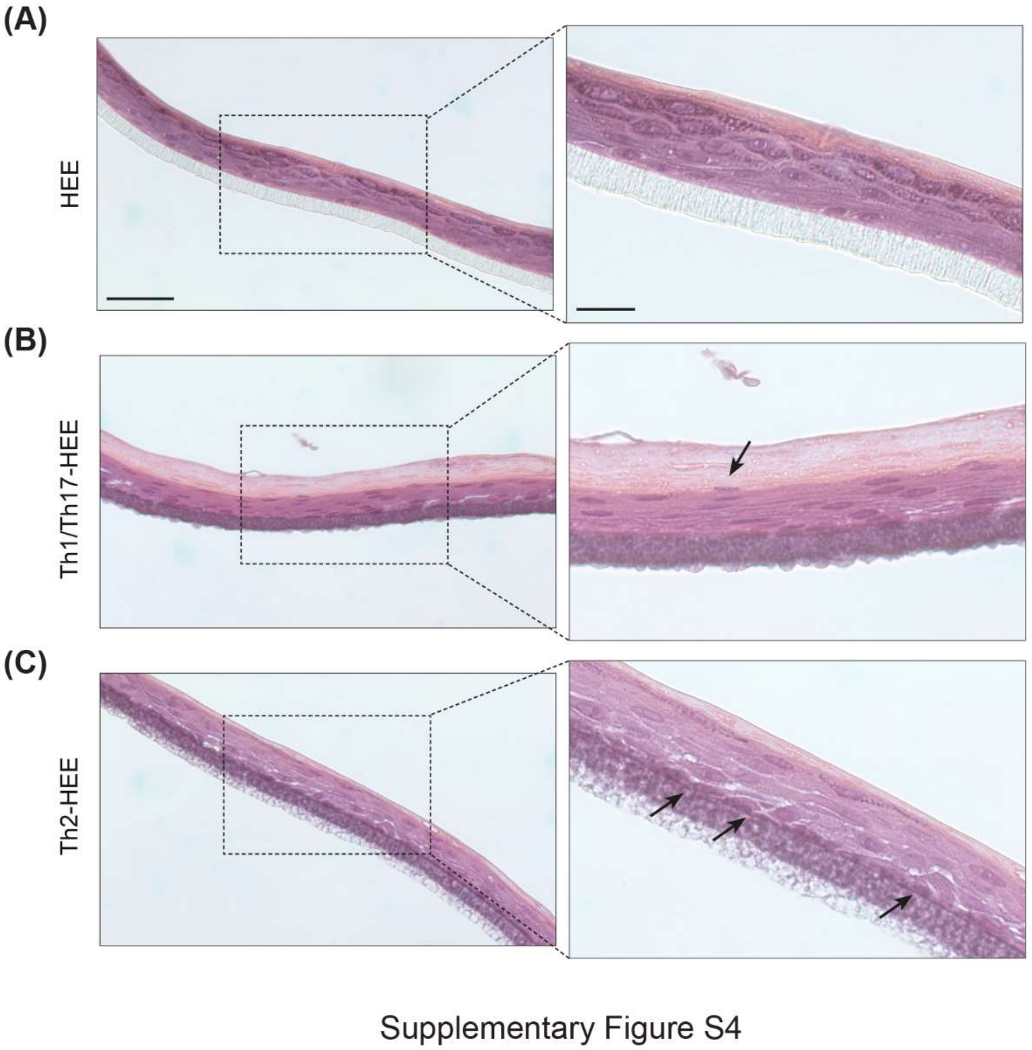

Supplementary Figure S4. Haematoxylin and eosin staining (H&E) of paraffin-embedded 3D HEEs, Th1/Th17-HEEs and Th2-HEEs. Histological analysis showed the presence of keratinocytes stratifying in multilayers from the basal to the granular layer and up to the outermost stratum corneum (A). Stimulation with Th1/Th17 cytokines induced the characteristic histological features of psoriasis such as loss of the stratum granulosum, thickening of the stratum corneum and parakeratosis (B, arrow). The addition of Th2 cytokines induced an AD phenotype-like as evidenced by the presence of intercellular white spaces between adjacent keratinocytes (C, arrows). Scale bars: 50 μm, 20 μm.

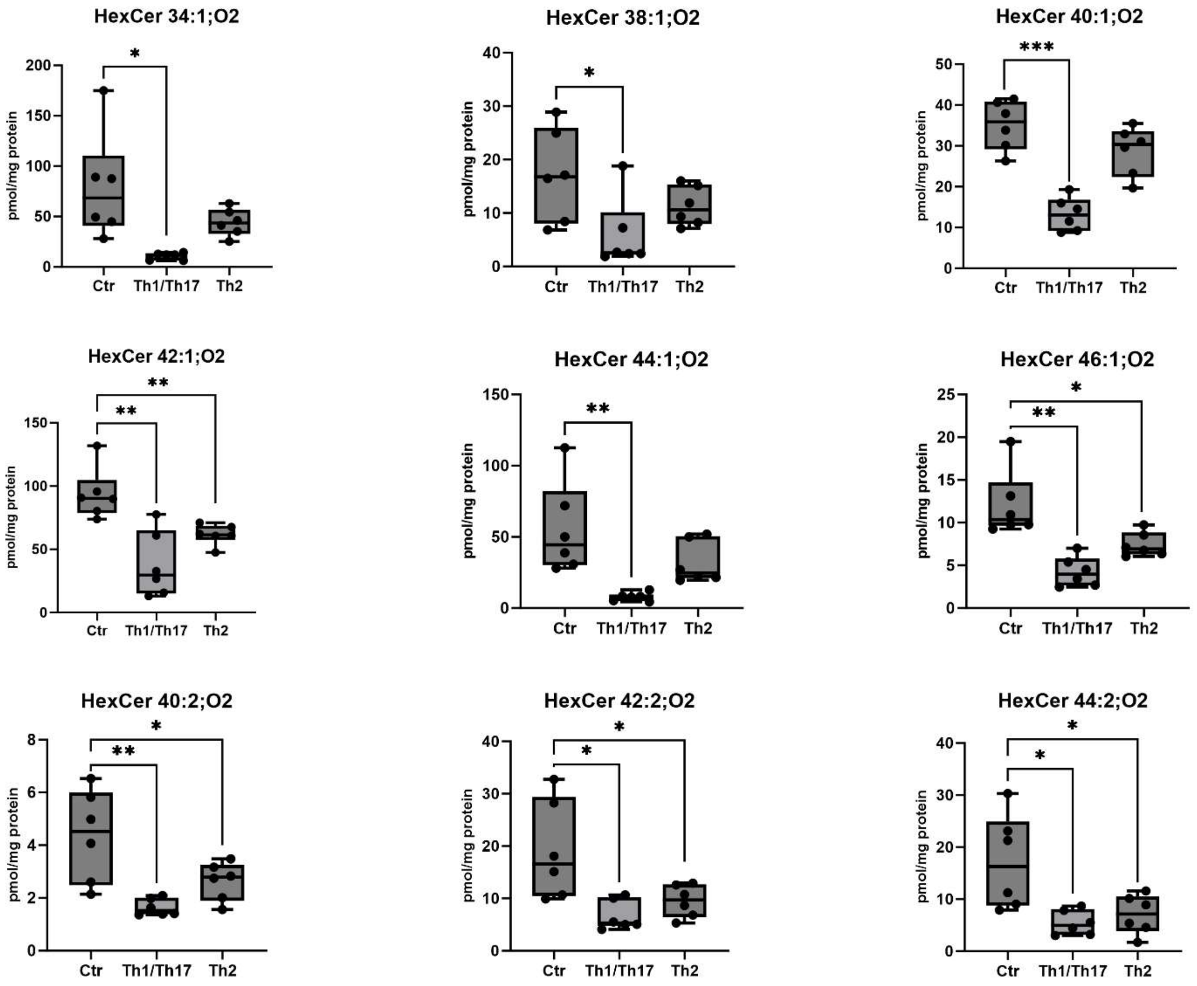

Supplementary Figure S5

Supplementary Figure S5.

Modification of the concentration (pmol/mg protein) of glucosylceramides, alias hexosylceramides (HexCers), reported in the heatmap (Figure 5) of 3D epidermal equivalents upon treatment with Th1/Th17 or Th2 cytokines. Notation of HexCers was assigned according to LIPIDMAPS nomenclature (https://www.lipidmaps.org/resources/education/classification). The box plots represent the data of three independent experiments.performed in duplicate. Significance vs control (Ctr) was marked with *p < 0.05, **p < 0.005 and ***p < 0.0005.
