# Supplementary material for: Effects of Th1/Th17 and Th2 cytokines on lipid metabolism in differentiated keratinocytes
Source: Front Physiol. 2025 Feb 19;16:1387128. doi: 10.3389/fphys.2025.1387128 (PMC11880217; doi:10.3389/fphys.2025.1387128)
Supplement: Supplementary file 1 [file DataSheet1.zip › Supplementary Data Sheet/Supplementary Table S1.docx]

| **330713 \| Deuterated Ceramide LIPIDOMIX®** | | | | |
| --- | --- | --- | --- | --- |
| **Compound name** | **Formula** | **Molecular weight** | **Exact mass** | **Conc µM** |
| C16 Ceramide-d7 (d18:1-d7/16:0) | C34H60D7NO3 | 544.94 | 544.5560 | 8 |
| C18 Ceramide-d7 (d18:1-d7/18:0) | C36H64D7NO3 | 573.00 | 572.5873 | 4 |
| C24 Ceramide-d7 (d18:1-d7/24:0) | C42H72D7NO3 | 657.16 | 652.6499 | 8 |
| C24:1 Ceramide-d7 (d18:1-d7/24:1(15Z)) | C42H74D7NO3 | 655.14 | 654.6656 | 4 |
| **330731 \| EquiSPLASH™ LIPIDOMIX®** | | | | |
| **Compound name** | **Formula** | **Molecular weight** | **Exact mass** | **Conc µM** |
| 15:0-18:1(d7) PC | C41H73D7NO8P | 753.11 | 752.6061 | 27 |
| 18:1(d7) Lyso PC | C26H45D7NO7P | 528.72 | 528.3921 | 38 |
| 15:0-18:1(d7) PE | C38H67D7NO8P | 711.03 | 710.5591 | 28 |
| 18:1(d7) Lyso PE | C23H39D7NO7P | 486.64 | 486.3451 | 41 |
| 15:0-18:1(d7) PG (Na Salt) | C39H67D7NaO10P | 764.02 | 763.5357 | 26 |
| 15:0-18:1(d7) PI (NH4 Salt) | C42H75D7NO13P | 847.13 | 846.5963 | 24 |
| 15:0-18:1(d7) PS (Na Salt) | C39H66D7NNaO10P | 777.02 | 776.5309 | 26 |
| 15:0-18:1(d7)-15:0 TG | C51H89D7O6 | 812.37 | 811.7646 | 25 |
| 15:0-18:1(d7) DG | C36H61D7O5 | 587.98 | 587.5506 | 34 |
| 18:1(d7) MG | C21H33D7O4 | 363.59 | 363.3366 | 55 |
| 18:1(d7) Chol Ester | C45H71D7O2 | 658.16 | 657.6441 | 30 |
| d18:1-18:1(d9) SM | C41H72D9N2O6P | 738.12 | 737.6397 | 27 |
| C15 Ceramide-d7 (d18:1-d7/15:0) | C33H58D7NO3 | 530.92 | 530.5404 | 38 |
| **In-house Standards MIX** | | | | |
| **Compound name** | **Formula** | **Molecular weight** | **Exact mass** | **Conc µM** |
| Hexadecanoic-d17 Acid (d17-PA) | C16H15D17O2 | 273.53 | 273.3469 | 80 |
| n-Hexadecyl-1,1,2,2-d4 Hexadecanoate-16,16,16-d3 (d17-PA) | C32H57D7O2 | 487.90 | 487.5346 | 40 |
| Glyceryl trihexadecanoate-d98 (d98TG 48:0) | C51D98O6 | 905.92 | 905.3515 | 20 |
| Deuterated cholesterol sulfate sodium salt (d7-CHS) | C27H38D7NaO4S | 495.34 | 495.3376 | 20 |
| N-palmitoyl-d31-D-erythro-sphingosine (d31-Cer16:0) | C34H37D30NO3 | 569.10 | 567.7004 | 1 |
| Deuterated cholesterol-2,2,3,4,4,6-d6 | C27H40D6O | 392.70 | 392.3925 | 40 |

**Supplementary Table S1.** List of the labelled internal standards used for extraction procedure. The final concentration added is expressed as µM.
