# Supplementary material for: Effects of Th1/Th17 and Th2 cytokines on lipid metabolism in differentiated keratinocytes
Source: Front Physiol. 2025 Feb 19;16:1387128. doi: 10.3389/fphys.2025.1387128 (PMC11880217; doi:10.3389/fphys.2025.1387128)
Supplement: Supplementary file 1 [file DataSheet1.zip › Supplementary Data Sheet/Supplementary Table S2.docx]

**Supplementary Table S2**

| Oligonucleotide sequences (5’-3’) | Amplicon size | Accession number |
| --- | --- | --- |
| **ABCA12**  sense: ACAGGAATGGCCTTCATCAC  antisense: AACATGGTGCCCTGAGAAAC | 317 bp | NM_015657.4 |
| **ALOX12B**  sense: GCTGCTGGTGCTCTGGAC  antisense: CTGGCGGATGTCGTGTGAG | 151 bp | NM_001139.3 |
| **CASP14**  sense: ACATCGCCTACCGACATGATC  antisense: CCGGGTCACCTCTGTCAGAA | 110 bp | NM_012114.3 |
| **CCL26**  sense: GCTGCTTCCAATACAGCCACA  antisense:TCCTTGGATGGGTACAGACTTTC | 135 bp | NM_001371936.1 |
| **CERS1**  sense: CAATGTGGGCATCCTTGTGCT  antisense: AGTAGAGGCGGAACCAGAAC | 176 bp | NM_001387443.1 |
| **CERS2**  sense: GCCAGGTAGAGCGTTGGTTC  antisense: GGCAATGAAGGCAATCAGGT | 113 bp | NM_181746.4 |
| **CERS3**  sense: TCTCTGCTGACTGCATCTATTG  antisense:GAAGCCAGAATCTTTCCAACC | 145 bp | NM_001290341.2 |
| **CERS4**  sense: GCAGTATCAGCAAGTGTGCG  antisense: CCTGTTGCTGATGGACTCGT | 124 bp | NM_024552.3 |
| **CERS5**  sense: TGACACCCTTTTTGTGATCTTCA  antisense: GAAGCATAAGGCCCGATTAT | 120 bp | NM_001331069.3 |
| **CERS6**  sense: GGGCGGACCTGAAGAACAC  antisense: CGCACGGTTTGGCTACAAAT | 129 bp | NM_203463.3 |
| **DEGS1**  sense: GTCTACACCGACCAGCCG  antisense: GCATAGGCCCCAAATATGACC | 188 bp | NM_001321541.2 |
| **DEGS2**  sense: GGTGCCTGTGCCCTACATTA  antisense: GGCCTCAAGCTCCACTCATC | 135 bp | NM_206918.3 |
| **ELOVL1**  sense: CCAAGGTCAAGGCCAACTGA  antisense: CTGACGGACACTGCCCTAAG | 87 bp | NM_001256401.2 |
| **ELOVL3**  sense: CAATGAAGCTCCAGGCTCTC  antisense: AACCATGCAGGTAAGGCAAC | 90 bp | NM_152310.3 |
| **ELOVL4**  sense: CCGGAATGGTCAAATCTCTCC  antisense: ACACCATCATCATCAAGCCTC | 111 bp | NM_022726.4 |
| **FADS2**  sense: TGTCTACAGAAAACCCAAGTGG  antisense: TGTGGAAGATGTTAGGCTTGG | 128 bp | NM_001281502 |
| **FAS**  sense: GACCGCTTCCGAGATTCC  antisense: GATGGCAGTCAGGCTCAC | 137 bp | NM_004104 |
| **FLG**  sense:GAAGACAAGGATCGCACCAG  antisense: ATGGTGTCCTGACCCTCTTG | 76 bp | NM_002016.2 |
| **GAPDH**  sense: TGCACCACCAACTGCTTAGC  antisense: GGCATGGACTGTGGTCATGAG | 198 bp | NM_001289746 |
| **HMGB1**  sense: GTGCCTCGCTGAGGAAAAAT  antisense: TCCTCCCGACAAGTTTGCAC | 105 bp | NM_001363661.2 |
| **HMGCR**  sense: AGCTACAATGTTGTCAAGAC  antisense: GCAGATGGTCAGTGTCAC | 97 bp | NM_000859.3 |
| **INV**  sense: ACCCATCAGGAGCAAATGAAA  antisense: GCTCGACAGGCACCTTCTGGC | 67 bp | NM_005547.4 |
| **K10**  sense: TGATGTGAATGTGGAAATGAATGC  antisense: GTAGTCAGTTCCTTGCTCTTTTCA | 147 bp | NM_000421.5 |
| **LOR**  sense: tcatgatgctacccgaggtttg  antisense: CAGAACTAGATGCAGCCGGAGA | 87 bp | NM_000427.3 |
| **SCD1**  sense: CATAATTCCCGACGTGGCTTT  antisense:AGGTTTGTAGTACCTCCTCTGGAACA | 150 bp | NM_005063 |
| **SLC27A4**  sense: AAATCGGGGAGTTCTACGGC  antisense: CAGGATGCGGCTGTTGAAAC | 95 bp | NM_198580.3 |
| **SPT**  sense: TGGTCATTTGGCCCAGGTC  antisense: TTCCAACCATTGGC TTCACATC | 121 bp | NM_004863.4 |
| **SREBP-1**  sense: GGAGCCATGGATTGCACTTT  antisense: TCAAATAGGCCAGGGAAGTCA | 77 bp | NM_001005291 |
| **TGM1**  sense: TCTTCAAGAACCCCCTTCCC  antisense: TCTGTAACCCAGAGCCT | 69 bp | NM_000359.3 |

**Supplementary Table S2.** Primers used for the Real time RT‐PCR analysis.
