# Supplementary material for: Effects of Th1/Th17 and Th2 cytokines on lipid metabolism in differentiated keratinocytes
Source: Front Physiol. 2025 Feb 19;16:1387128. doi: 10.3389/fphys.2025.1387128 (PMC11880217; doi:10.3389/fphys.2025.1387128)
Supplement: Supplementary file 1 [file DataSheet1.zip › Supplementary Data Sheet/Supplementary Table S4.docx]

| **LIPID METABOLISM** | | | | | | | | | |
| --- | --- | --- | --- | --- | --- | --- | --- | --- | --- |
|  | **2 days** | | | **4 days** | | | **7 days** | | |
|  | **↑Ca^2+^** | **↑Ca^2+^ +**  **Th1/Th17** | **↑Ca^2+^**  **+ Th2** | **↑Ca^2+^** | **↑Ca^2+^ +**  **Th1/Th17** | **↑Ca^2+^**  **+Th2** | **↑Ca^2+^** | **↑Ca^2+^ +**  **Th1/Th17** | **↑Ca^2+^**  **+Th2** |
| ***ABCA12*** | 1,13±0,26 | 1,18±0,27 | 1,08±0,30 | **2,13±0,35** | **2,01±0,40** | **1,99±0,32** | **7,29±0,70** | **3,84±0,50** | **4,93±0,70** |
| ***ALOX12B*** | **1,65±0,45** | **1,57±0,38** | **1,67±0,40** | **3,01±0,50** | **4,91±0,60** | **4,79±0,56** | **6,02±0,60** | **11,29±0,90** | **10,6±0,85** |
| ***CERS1*** | 1,03±0,38 | 1,44±0,35 | 1,47±0,11 | **2,45±0,38** | **2,47±0,08** | **4,34±0,27** | **3,81±0,14** | **2,35±0,27** | **6,93±3,00** |
| *CERS2* | 1,23±0,09 | 1,03±0,11 | 0,90±0,08 | 0,98±0,08 | 1,03±0,07 | 0,93±0,09 | 0,93±0,09 | 1,28±0,11 | 1,07±0,11 |
| ***CERS3*** | 1,39±0,23 | **1,58±0,20** | 1,50±0,16 | **1,72±0,19** | **1,82±0,16** | **2,20±0,23** | **1,72±0,16** | **4,01±0,27** | **2,41±0,14** |
| ***CERS4*** | 1,09±0,16 | 1,23±0,009 | 1,02±0,11 | **1,88±0,15** | **2,23±0,18** | **2,42±0,23** | **3,86±0,60** | **3,28±0,14** | **3,56±0,33** |
| ***CERS5*** | 0,98±0,06 | 0,92±0,08 | 0,77±0,08 | 0,91±0,11 | 0,92±0,08 | 0,90±0,12 | **1,54±0,11** | 1,41±0,13 | **1,67±0,12** |
| ***CERS6*** | 1,13±0,11 | 0,84±0,13 | 1,04±0,10 | 1,23±0,12 | 1,04±0,13 | 1,22±0,14 | **3,11±0,25** | 1,32±0,17 | **2,34±0,18** |
| ***DEGS1*** | 0,87±0,12 | 1,15±0,09 | 1,02±0,08 | 1,08±0,09 | 0,91±0,10 | 0,86±0,09 | **1,77±0,09** | 1,18±0,12 | **2,18±0,12** |
| ***DEGS2*** | 1,20±0,68 | 0,82±0,15 | **2,11±0,75** | **2,54±1,42** | **1,68±0,14** | **2,76±0,61** | **4,59±0,96** | **2,66±0,05** | **12,89±2,08** |
| *ELOVL1* | 0,99±0,08 | 1,10±0,07 | 0,97±0,09 | 1,21±0,10 | 0,95±0,09 | 1,09±0,08 | 0,97±0,11 | 0,99±0,08 | 1,10±0,09 |
| ***ELOVL3*** | 0,51±0,25 | 0,43±0,10 | 0,43±0,35 | 1,22±0,21 | 0,40±0,13 | 0,83±0,14 | **4,16±0,69** | **2,08±0,26** | **4,97±0,40** |
| *ELOVL4* | 1,01±0,11 | 0,92±0,12 | 1,28±0,08 | 1,28±0,13 | 0,93±0,14 | 1,35±0,12 | 1,15±0,09 | 1,22±0,11 | 1,36±0,10 |
| *FADS* | 1,12±0,09 | 1,14±0,10 | 1,12±0,08 | 1,13±0,12 | 1,08±0,08 | 1,03±0,11 | 0,64±0,05 | 0,78±0,08 | 0,88±0,03 |
| *FAS* | 0,98±0,08 | 1,06±0,09 | 1,19±0,10 | 1,10±0,12 | 1,20±0,08 | 1,00±0,09 | 0,63±0,04 | 0,72±0,04 | 0,88±0,08 |
| ***HMGB1*** | 0,97±0,10 | 0,84±0,12 | 1,08±0,09 | 1,09±0,12 | 0,90±0,11 | 0,78±0,13 | **2,12±0,13** | 1,38±0,10 | 1,36±0,12 |
| ***HMGCR*** | 1,17±0,11 | 0,82±0,09 | 1,31±0,11 | 1,03±0,12 | 1,03±0,09 | 1,22±0,10 | **1,56±0,08** | 1,16±0,10 | 1,32±0,08 |
| *SCD* | 0,99±0,07 | 0,77±0,08 | 1,08±0,06 | 0,92±0,02 | 0,96±0,04 | 0,90±0,10 | 0,57±0,05 | 0,82±0,05 | 0,79±0,07 |
| ***SLC27A4*** | 1,04±0,10 | 0,96±0,09 | 0,84±0,10 | 0,91±0,08 | 1,00±0,11 | 0,93±0,06 | **1,99±0,10** | **1,73±0,08** | **1,57±0,09** |
| *SPT* | 0,93±0,12 | 0,97±0,11 | 1,11±0,13 | 1,05±0,14 | 1,05±0,10 | 1,00±0,09 | 0,98±0,12 | 1,18±0,11 | 0,85±0,12 |
| *SREBP1* | 1,09±0,10 | 1,02±0,12 | 0,82±0,09 | 0,89±0,11 | 0,91±0,12 | 0,88±0,08 | 0,85±0,09 | 1,09±0,10 | 1,00±0,12 |

**Supplementary Table S4.** Mean values and standard deviation (SD) of fold changes (FC) of mRNA expression of lipid metabolism genes in Ker CT cells treated with high calcium (↑Ca^2+^), high calcium together with Th1/Th17 cytokines (↑Ca^2+^ + Th1/Th17) and high calcium with Th2 cytokines (↑Ca^2+^ + Th2) at 2,4 and 7 days compared to vehicle. Genes that presented statistically significant differences (p≤0,05) are indicated in bold.
